# High expression of the underexplored *SLC4A11* protein-coding transcript is specific to the corneal endothelium

**DOI:** 10.1038/s41598-026-51987-w

**Published:** 2026-05-11

**Authors:** Elena S. Kotova, Elena I. Sharova, Polina A. Kovaleva, Boris E. Malyugin, Olga P. Antonova, Alexandra V. Belodedova, Ivan S. Tkachenko, Yuriy V. Doludin, Tatiana N. Garmanova, Mikhail Protasov, Tatiana R. Tsedilina, Liubov O. Skorodumova

**Affiliations:** 1 Lopukhin Federal Research and Clinical Center of Physical-Chemical Medicine of Federal Medical Biological Agency, Malaya Pirogovskaya, 1a, Moscow, 119435 Russia; 2 The Jules Stein Eye Institute, University of California at Los Angeles, 200 Stein Plaza, Los Angeles, CA 90095 USA; 3 Department of Anterior Segment Transplant and Optical Reconstructive Surgery, S. Fyodorov Eye Microsurgery Complex Federal State Institution, Beskoudnikovsky Boulevard, 59A, Moscow, 127486 Russia; 4 National Medical Research Center for Therapy and Preventive Medicine, Petroverigskyj Lane 10, bld.3, Moscow, 101990 Russia; 5 Lomonosov Moscow State University, Lomonosovskiy Prospekt, 27 bld.10, Moscow, 119192 Russia

**Keywords:** *SLC4A11*, Corneal endothelium, Fuchs corneal dystrophy, Transcription initiation, Congenital hereditary endothelial dystrophy, Transcription, Transcriptional regulatory elements, Corneal diseases, Mechanisms of disease

## Abstract

Fuchs’ endothelial corneal dystrophy (FECD) and congenital hereditary endothelial dystrophy (CHED) are corneal endothelial pathologies associated with *SLC4A11* gene variants. The principal findings on the expression and function of *SLC4A11* in the corneal endothelium have been based on studies of three major transcripts variants (v1, v2 and v3) and their protein products. We aimed to address gaps and resolve contradictions regarding the expression of *SLC4A11*. Our transcriptomic analysis revealed predominant expression of RefSeq-annotated protein-coding transcripts v6 and v3, but not v2 and v1, in the corneal endothelium of patients with FECD and controls. We also demonstrated the specificity of high *SLC4A11* and major transcript expression to corneal endothelium. Using the 5’ RACE method, we directly showed that the major transcription start site is at the beginning of v6, from which v3 with an extended 5’UTR is also transcribed. We estimated the fraction of v6 and v3 from the overall expression as 0.63 and 0.34, respectively. We also obtained evidence of full-length v6 expression in corneal endothelium and its ability to drive translation of the previously described v2M36 protein isoform. These findings provide new information on *SLC4A11* gene expression and functional consequences of some genomic variants identified in patients with FECD.

**Supplementary Information:**

The online version contains supplementary material available at 10.1038/s41598-026-51987-w.

## Introduction

*SLC4A11* gene encodes an ion transporter that likely plays a role in maintaining corneal stromal water homeostasis^1^. Single- and multinucleotide variants in this gene were implicated in development of corneal dystrophies: congenital hereditary endothelial dystrophy (CHED) and primary Fuchs’ endothelial corneal dystrophy (FECD). FECD is an inherited disorder characterized by a progressive reduction in corneal endothelial cells, which are crucial for maintaining the water balance in the corneal stroma. FECD is a multigenic disease, with the most prevalent causative variant (approximately 70%) in populations of European descent being the CTG18.1 trinucleotide repeat expansion in the *TCF4* gene^2,3^. Additionally, heterozygous single nucleotide variants in the *SLC4A11* gene have also beenproposed as causal for FECD^4,5^. A systematic review reported that variants of uncertain significance (VUS), as well as likely pathogenic or pathogenic *SLC4A11* variants, were found in 2.5% of FECD patients across case-control and case-series studies reported between 2005 and 2022^6^. Notably, the majority of these variants were detected in Indian and Chinese cohorts, populations characterised by a low to moderate frequency (17–44%) of CTG18.1 expansion^7^. A recent study of FECD patients from the UK and the Czech Republic identified rare and potentially pathogenic *SLC4A11* variants in only 2 out of 128 participants (1.6%) who lacked the CTG18.1 expansion^8^. Thus, heterozygous variants in the *SLC4A11* gene contribute to a small but notable proportion of FECD cases. Homozygous or compound heterozygous mutations in the *SLC4A11* gene are associated with approximately 80% of congenital hereditary endothelial dystrophy (CHED) cases^9–11^. Conflicting reports exist regarding *SLC4A11* expression in FECD patients, with some studies showing decreased *SLC4A11* expression^4^ and others showing increased *SLC4A11* expression^12,13^. These discrepancies may be due to variations in the FECD stages^13^.

Understanding the role of *SLC4A11* in the pathogenesis of endothelial dystrophies and the functional analysis of potentially pathogenic variants requires identification of the most abundantly expressed protein-coding transcript and the dominant protein isoform. However, data on the most abundant transcripts in the corneal endothelium are contradictory. To date, much of the literature on the function of the SLC4A11 protein product in the corneal endothelium has been based on the existence of three major transcript variants that differ in their 5’ ends and have different N-terminal fragments of their encoded proteins. In 2001, the *SLC4A11* transcript was first identified within renal RNA^14^. The corresponding transcript variant v2 (NM_032034) is recorded in the NCBI RefSeq database. Until 2015, it was believed that this transcript represented the predominant *SLC4A11* variant in the corneal endothelium,^5,11^ with its protein product initiating translation from the first ATG. Kao et al. published the first study in 2015 analyzing not only isoform 2 but also isoforms 1 (corresponding to transcript variant v1, NM_001174090) and 3 (corresponding to transcript variant v3, NM_001174089) of *SLC4A11*^15^. Some studies have shown that the expression levels of transcripts 2 and 3 in the corneal endothelium are close to each other^16,17^. However, Kao et al. reported that v3 is the most highly expressed variant and that both v3 and v1 (NM_001174090) are more abundant than v2 in the corneal endothelium^18^.

For the functional analysis of variants associated with corneal dystrophies, constructs expressing proteins encoded by v2 and v3 are utilized^17,19–21^. Additionally, evidence has shown that the major isoform of the SLC4A11 protein in the corneal endothelium is an N-short polypeptide, hereafter referred to as v2M36^17^. v2M36 has been suggested to result from the translation of v2 starting not from the first but from the second ATG codon in the same reading frame. However, some papers continue to focus on the functional characterization of the full-length protein encoded by v2 rather than that encoded by v2M36.^21^ Consequently, additional data is needed to clarify the primary *SLC4A11* transcript and protein isoform in corneal endothelium.

In most studies, the prevalence of a particular transcript variant in the corneal endothelium has been determined by real-time PCR using primers for regions that differ between v1, v2 and v3^17,18^. However, to date, no attempts to obtain a comprehensive set of *SLC4A11* transcripts present in the corneal endothelium or, at least, to characterize the set of the most represented 5’ ends of mRNAs, which are different from the known *SLC4A11* protein-coding transcripts, have been made. Additionally, identifying the most abundant 5’ ends of *SLC4A11* transcripts in the corneal endothelium would provide further insights into the function of the promoter region of this gene. According to bioinformatic analysis, the potential promoter of *SLC4A11* is located significantly (more than one kbp) upstream of the 5’ end of v2.^22,23^ However, the transcription start sites (TSSs) of the *SLC4A11* gene were not identified experimentally in the corneal endothelium. Obtaining more detailed information regarding location and functioning of *SLC4A11* promoter elements is important, as it may illuminate the mechanisms underlying the pathogenesis of FECD and CHED.

Upon visualization of read coverage in the *SLC4A11* gene locus using published transcriptomes (total and poly-A) in the corneal endothelium, we observed some inconsistencies between the transcriptomic data and the literature. Consequently, we obtained a comprehensive set of *SLC4A11* transcripts that may be present in endothelial transcriptomes. Next, we analyzed the expression of alternative transcript variants in the corneal endothelium and in different tissues. Additionally, we localized the main TSS of *SLC4A11* in the corneal endothelium of healthy subjects and patients with FECD.

## Results

### Inconsistencies in the data regarding the most highly expressed protein-coding transcripts of *SLC4A11*

**Fig. 1:**
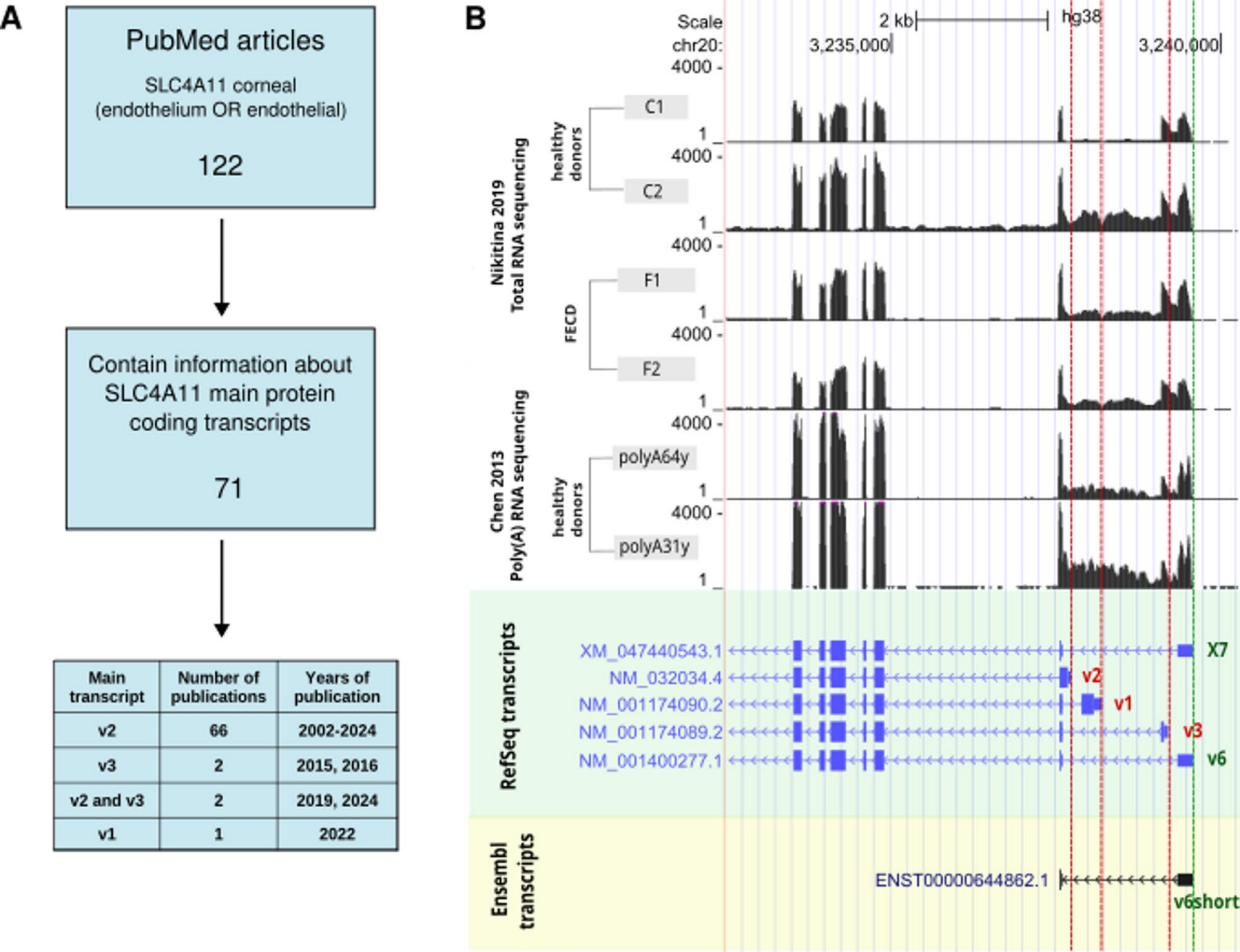
Inconsistency in evidence for the most represented protein-coding transcript variant of *SLC4A11*. (**A**) The results of the literature analysis showing the number of articles found. Currently, transcript 2 appears in the largest number of articles as the most highly expressed transcript. (**B**) RNA-seq read density in the 5’-end region of *SLC4A11*. The names of the transcript variants discussed and not mentioned in the literature are red and green, respectively.

To determine which transcript variants are most abundant in the corneal endothelium, we searched PubMed for articles discussing *SLC4A11* transcripts or corresponding protein isoforms. We formulated the query “SLC4A11 corneal (endothelial OR endothelium)” to maximize the breadth of our search (Fig. 1A, Supplementary File 1). A total of 122 articles were retrieved and analyzed. We excluded 15 review articles, one correction, and two articles lacking English text. This left us with 33 publications that did not provide information on the transcript variants of the *SLC4A11* gene in the human corneal endothelium and 71 original studies with relevant information on *SLC4A11* transcript variants. Among these 71 articles, 68 identified transcript variant 2 as the major transcript (66 publications) or one of the major transcripts (two publications). Additionally, two articles considered v3 to be the major transcript and one article used v1 for mutation annotation. Notably, the articles reviewed did not reference other protein-coding transcripts of *SLC4A11* in the corneal endothelium. Four articles reported that v2M36 is the major isoform of the SLC4A11 protein.

To further refine the information obtained from the literature, we visualized read coverage in the *SLC4A11* gene locus using published transcriptomes (four total, two poly-A) of corneal endothelium from healthy donors and patients with FECD^24,25^ (Fig. 1B). Besides v1, v2 and v3, other transcript variants capable of encoding known SLC4A11 protein isoforms are present in the RefSeq and Ensembl databases. These transcripts (including v1, v2 and v3) differ in their 5’-end regions. Across all samples, the 5’ region (first exon) of transcript variant v6 (NM_001400277; RefSeq) showed higher read coverage than those of v1, v2 and v3. In the RefSeq database, v6 is annotated as protein-coding, with a protein sequence (NP_001387206.1) identical to v2M36, which has been recently reported^17^ as the major protein product of v2 and the principal SLC4A11 protein isoform in the corneal endothelium. However, it is important to consider that the 5’-region of transcript 6 also belongs to the predicted transcript XM_047440543 (variant X7) from RefSeq, as well as the short transcript ENST00000644862 from Ensembl. The transcript variant ENST00000644862 is fully incorporated into v6 but lacks most of the SLC4A11 reading frame. Henceforth, we refer to it as v6short.

The density of reads corresponding to the first exon of v3 was similar to that of the first exon of v6 in most samples. However, the 5’ regions of v1 and v2 presented a lower read density, approaching background levels in some samples (samples C1 and F1, Fig. 1B). Therefore, we found inconsistencies between published and experimental data concerning *SLC4A11* transcripts in the corneal endothelium, which requires refinement.

### Estimation of *SLC4A11* transcript variant expression in the corneal endothelium

To resolve the discrepancies regarding which transcript variant of *SLC4A11* is predominant in the corneal endothelium, we first estimated the expression of different transcript variants in corneal endothelial transcriptomes. We utilized the two large published sets of corneal endothelial transcriptomes to characterize *SLC4A11* transcript expression. The extended Nikitina et al.^24^ transcriptome dataset (22 samples) will be referred to as endo1. The dataset from the publication by Chu et al.^26^ (25 samples) will be referred to as endo2. Details about datasets are described in the Materials and methods section and Supplementary File 2.

To compile the most comprehensive list of potential *SLC4A11* transcripts, we combined 27 unique *SLC4A11* transcripts represented in RefSeq and Emsembl databases. To ensure that our assessment of *SLC4A11* transcript expression was as complete as possible, we supplemented the obtained annotation file with two de novo-assembled transcripts. We propose that these two novel transcript variants lack significant protein-coding potential. One of them (MSTRG.58852.10, Supplementary Files 3 and 4) terminates before the last exon‒exon junction, which should lead to a nonsense-mediated decay (NMD)-dependent reduction in transcript abundance. The second variant (MSTRG.58852.21, Supplementary Files 3 and 4) has most of the *SLC4A11* reading frame truncated.

**Fig. 2:**
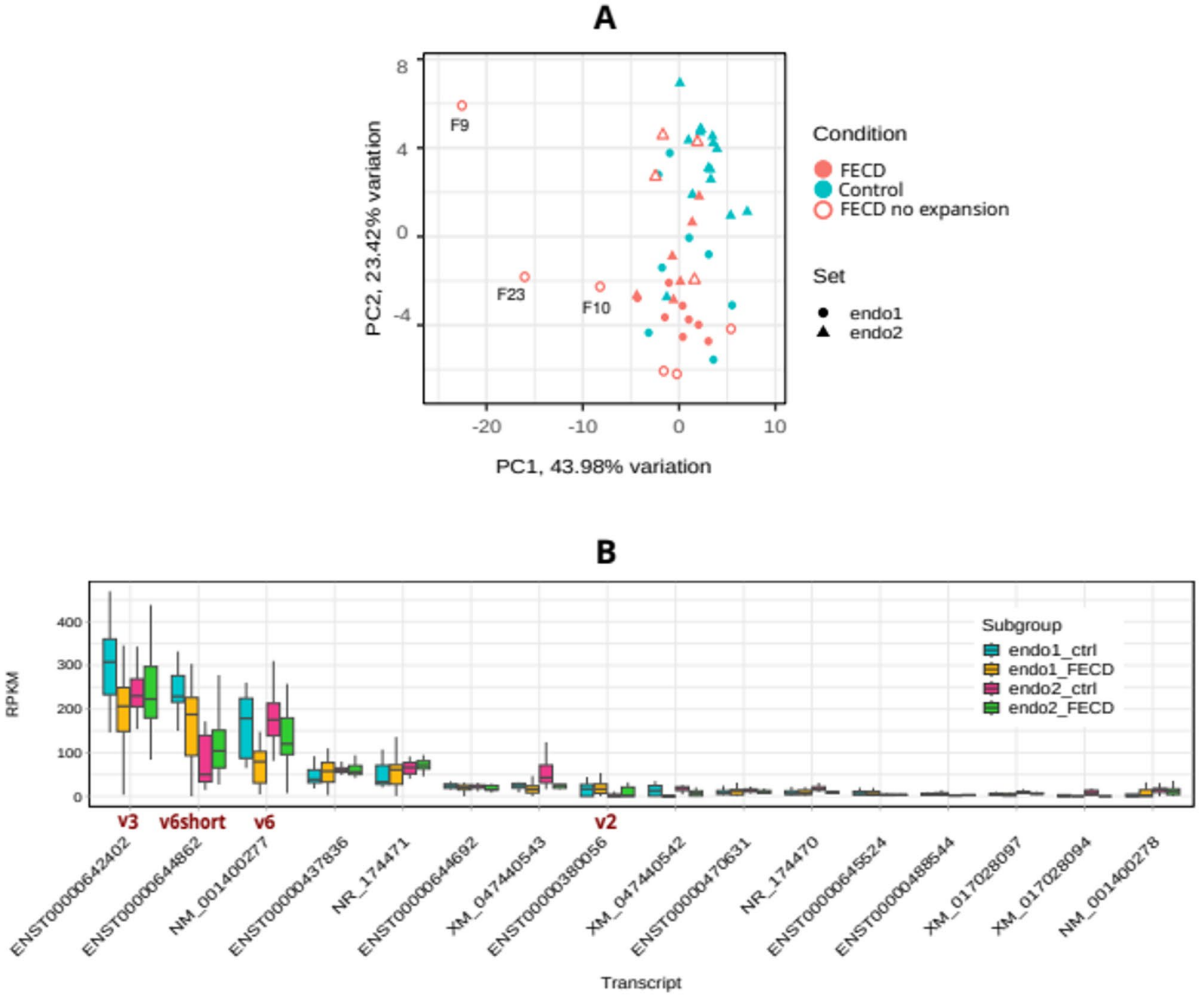
Analysis of *SLC4A11* transcript expression in two sets of corneal endothelium transcriptomes. (**A**) Lack of clear differences in the expression level of *SLC4A11* transcripts between the FECD and control groups in the two sets of corneal endothelium transcriptomes (PCA biplot on *SLC4A11* transcript expression levels in log2RPKM). (**B**) *SLC4A11* transcript expression in corneal endothelium datasets. The transcript variants whose maximal RPKM across all the samples was lower than 6 are not shown as expressed at low levels. Endo1_ctrl and endo2_ctrl are the control groups of the endo1 and endo2 datasets, respectively. Endo1_FECD and endo2_FECD are the FECD groups of the endo1 and endo2 datasets, respectively. Transcripts ranged by the median number of transcripts in the control group of the endo1 dataset.

Earlier Chu et al.^26^ found that transcriptomic data variability is influenced primarily by FECD status. We observed clear separation of the control and FECD sample groups in the principal component analysis (PCA) biplot for both datasets that aligned with previous data (Supplementary Figure S1). Analysis of sample heterogeneity across both datasets, based on *SLC4A11* transcript variant expression levels, revealed no separation between control and FECD groups in the PCA biplot (Fig. 2A). Therefore, FECD status does not appear to be the main factor influencing the variability of *SLC4A11* transcript variant expression in the samples studied.

*SLC4A11* transcript abundances in the FECD and control groups of both datasets (endo1 and endo2) are shown in Fig. 2B. Transcript v3 was identified as the most represented transcript in both endothelial datasets and across both groups classified by FECD status (Fig. 2B). The five most highly expressed transcripts also included the short and long forms of v6, ENST00000437836 (hereafter referred to as E_7836), and NR_174471 in both datasets, regardless of FECD status. Notably, v2, which is frequently cited in the literature as the major protein-coding transcript in the corneal endothelium, ranked only eighth among transcripts on the basis of RPKM in the control group of endo1. Compared with those of v2, the expression levels of each of the five most highly expressed transcript variants were significantly different within the same group and dataset (Wilcoxon test, p < 0.01; Supplementary Table S2). Among all the samples from both datasets, only one sample presented a nonzero RPKM for transcript 1. Therefore, we propose that this RNA isoform, which some articles refer to as the main *SLC4A11* protein-coding transcript, is expressed at a very low level in the corneal endothelium. Among the five most represented *SLC4A11* transcripts, only v3 and v6 in consistency with RefSeq and our ORF analysis by TransDecoder correspond to the currently investigated isoforms of this protein: 3 and v2M36, respectively^17^. NR_174471 is classified as a noncoding RNA in RefSeq and has several upstream open reading frames (ORFs) in its long 5’ UTR preceding the ORF of v2. Variants v6short and E_7836 are short RNA isoforms with truncated ORFs, comprising a portion (less than half) of the ORF coding for v2M36 and lacking a stop codon. Their potential protein products, with only 10 and 104 amino acids, would be structurally and functionally distinct from a typical SLC4A11 protein with membrane localization. No significant differences (Wilcoxon test, significance level of 0.01, Supplementary Table S3) were detected in the expression of the five most represented transcripts or v2 between the FECD and control groups in either dataset. This finding aligns with the lack of separation between the FECD and control groups in the PCA biplot of *SLC4A11* transcript expression levels (Fig. 2B). Interestingly, three samples from the FECD group of the endo1 set, without expansion of CTG18.1 repeats in the *TCF4* gene, were separated from the majority of samples by principal component 1 (Fig. 2B). These samples presented lower expression of *SLC4A11* than the other samples did.

### *SLC4A11* transcript variant expression in different tissues

To determine whether the preferential expression of SLC4A11 isoforms 3 and 6 in corneal endothelium is tissue-specific, we analyzed nine transcriptomic datasets (see Materials and methods). In addition to thecorneal endothelial datasets (endo1 and 2), these included datasets for thyroid^27^(“thyr”), and salivary gland^28^(“saliv”)—tissues with the highest *SLC4A11* expression per GTEx data^29^—using 10 randomly selected samples each. Further datasets comprised normal colon organoids (“col_org1"^30^, *n* = 5 and “col_org2"^31^, *n* = 12 ), corneal stroma/epithelium fragments from 8 healthy donors and 10 keratoconus patients (“kc”; our laboratory data), B-cell blood cancers^32^(“lymph”; *n* = 4), and colorectal cancers (“col_can”; *n* = 6; forthcoming publication). Colon and corneal stroma was selected due to reported *SLC4A11* expression^29,33^, and tumors given *SLC4A11*’s role in glutamine-addicted tumors^34^.

**Fig. 3:**
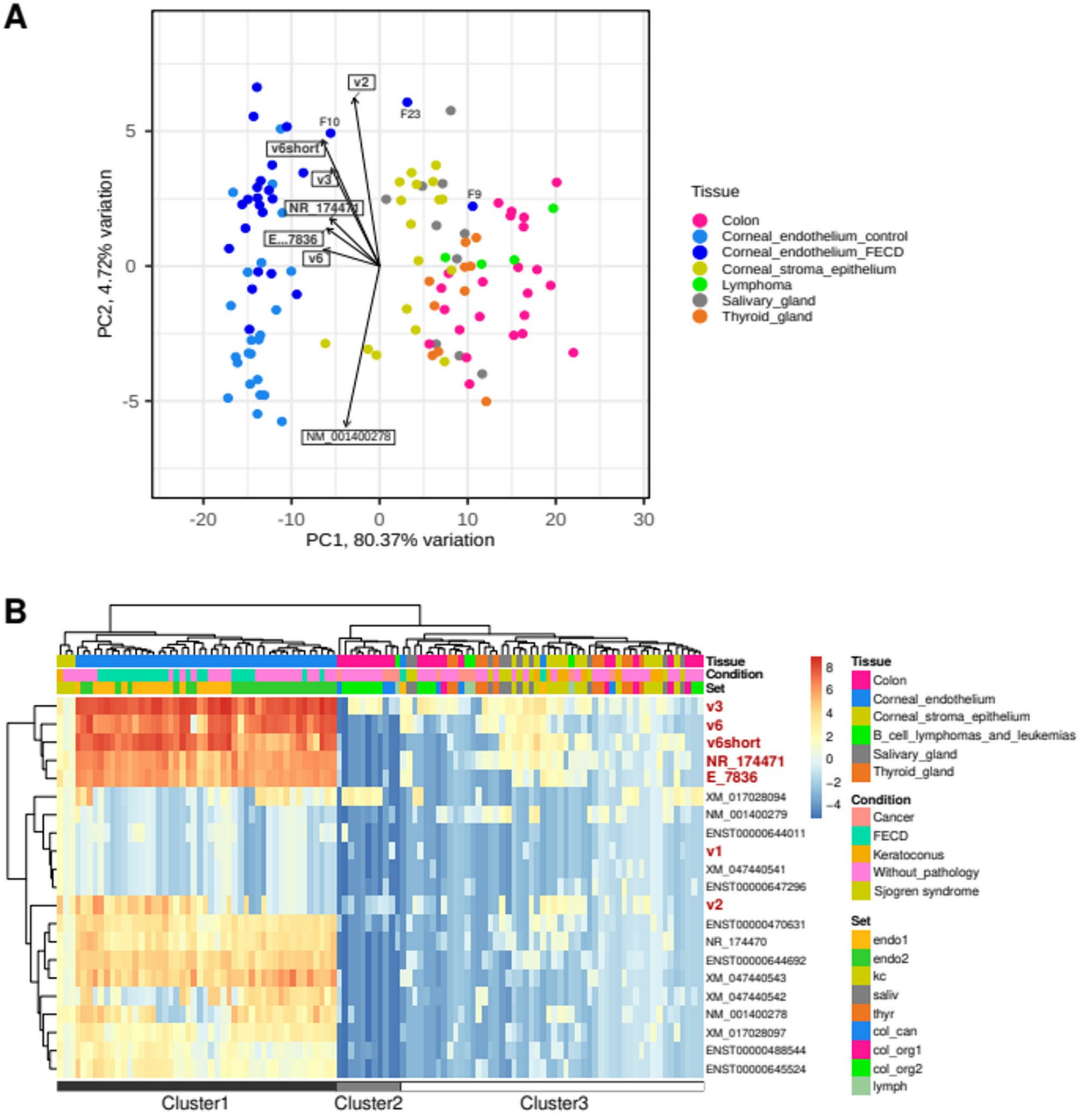
Expression of *SLC4A11* transcripts in samples of different tissues from local and external datasets. The analyzed transcriptomic datasets corresponded to the following tissues: corneal endothelium (endo1 and endo2), corneal fragments that consisted of stroma or stroma and epithelium but did not contain endothelium (kc), salivary gland (saliv), thyroid gland (thyr), colon (col_can, col_org1, col_org2), B-cell lymphomas and leukemias (lymph). (**A**) PCA of *SLC4A11* transcript expression levels (log2 RPKM) in samples of different tissues. The loading vectors for the transcripts used to generate the principal components are shown. (**B**) Heatmap of *SLC4A11* transcript variant expression in the analyzed samples. The transcripts and samples are clusterized. The main clusters of the samples are marked as Clusters 1–3. The names of the five most abundant transcripts in the corneal endothelium according to the transcriptome analysis and the two transcripts reported in the literature as major *SLC4A11* transcripts are marked in red.

The heterogeneity of the samples in these datasets was assessed through PCA based on *SLC4A11* transcript variant expression levels (Fig. 3A). The vast majority of corneal endothelial samples were separated from other tissue samples by principal component 1, indicating that much of the variance is related to whether the tissue is corneal endothelium or not. Among the top seven transcripts contributing to the principal components, five presented the highest expression within the corneal endothelium. Notably, samples F9 and F23 from the endo1 dataset were separated from the other corneal endothelial samples by PC1. These samples, along with F10 sample, had no trinucleotide repeat expansions in the *TCF4* gene and were positioned separately from other endothelial samples on the PCA biplot shown above (Fig. 2A). Sample F10 colocalized with the other endothelial samples shown in Fig. 3A but was the closest to F9 and F23.

The *SLC4A11* transcript expression levels in the analyzed samples were clustered by transcript and sample, as presented in the heatmap (Fig. 3B).

Three main sample clusters were evident in the heatmap. The first contained most of the endothelial samples from both datasets, whereas the second was primarily represented by colon samples. *SLC4A11* transcripts were also divided into three major clusters. The five transcripts most highly expressed in the corneal endothelium coclustered together (v3, v6, v6short, E_7836, and NR_174471). Among these, only v3 was upregulated in samples from cluster 2. The expression of the XM_017028094 transcript variant was also elevated in most (7 of 10) samples from cluster 2, in contrast with the majority of the corneal endothelial samples. Notably, this transcript is a potential protein-coding transcript whose reading frame corresponds to the v2M36 protein, similar to the reading frame of v6. Therefore, we observed that a subset of the analyzed samples displayed a different expression pattern of *SLC4A11* transcript variants than did the corneal endothelial samples.

**Fig. 4:**
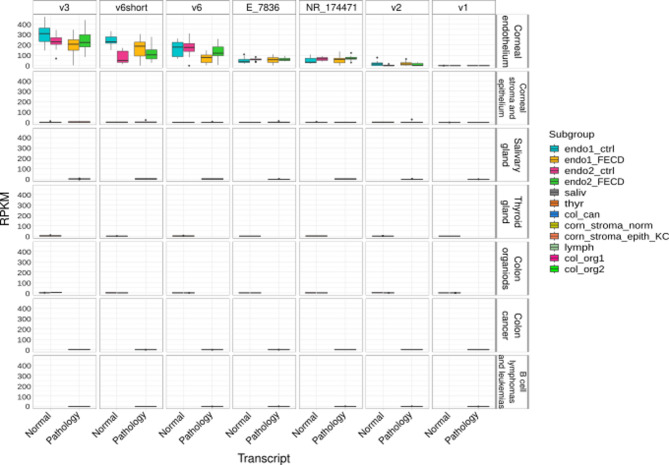
The expression levels of the *SLC4A11* transcripts were the highest in the corneal endothelium in samples combined by tissue, set and the presence or absence of pathology. The following subgroups of samples are shown: endo1_ctrl, control group of the endo1 set; endo1_FECD, FECD group of the endo1 set; endo2_ctrl, control group of the endo2 set; endo2_FECD, FECD group of the endo2 set; saliv, samples from the saliv set; thyr, samples from the thyr set; col_can, samples from the col_can set; corn_stroma_norm, normal corneal stroma from the kc set; corn_stroma_epith_KC, corneal stroma and corneal stroma with epithelium from patients with keratoconus from the kc set; lymph, samples from the lymph set; col_org1, samples from the col_org1 set; and col_org2, samples from the col_org2 set.

The expression levels of *SLC4A11* transcripts, which are most represented in the corneal endothelium according to our transcriptomic analysis, in groups of samples categorized by tissue, dataset, and the presence or absence of pathology, are illustrated in Fig. 4. The expression levels of v3, v6short, v6, E_7836, and NR_174471 in the corneal endothelium were considerably greater than those of the same *SLC4A11* transcripts in any other tissue type (Fig. 4). We also compared the proportions of *SLC4A11* transcripts among the analyzed datasets (Supplementary Figure S3). Although transcripts v3, v6, and v6short exhibited similarly high proportions of total *SLC4A11* expression in the corneal endothelial sample groups, some sample groups, such as col_org2 and lymph, expressed v3 but did not express v6 or v6short.

**Fig. 5:**
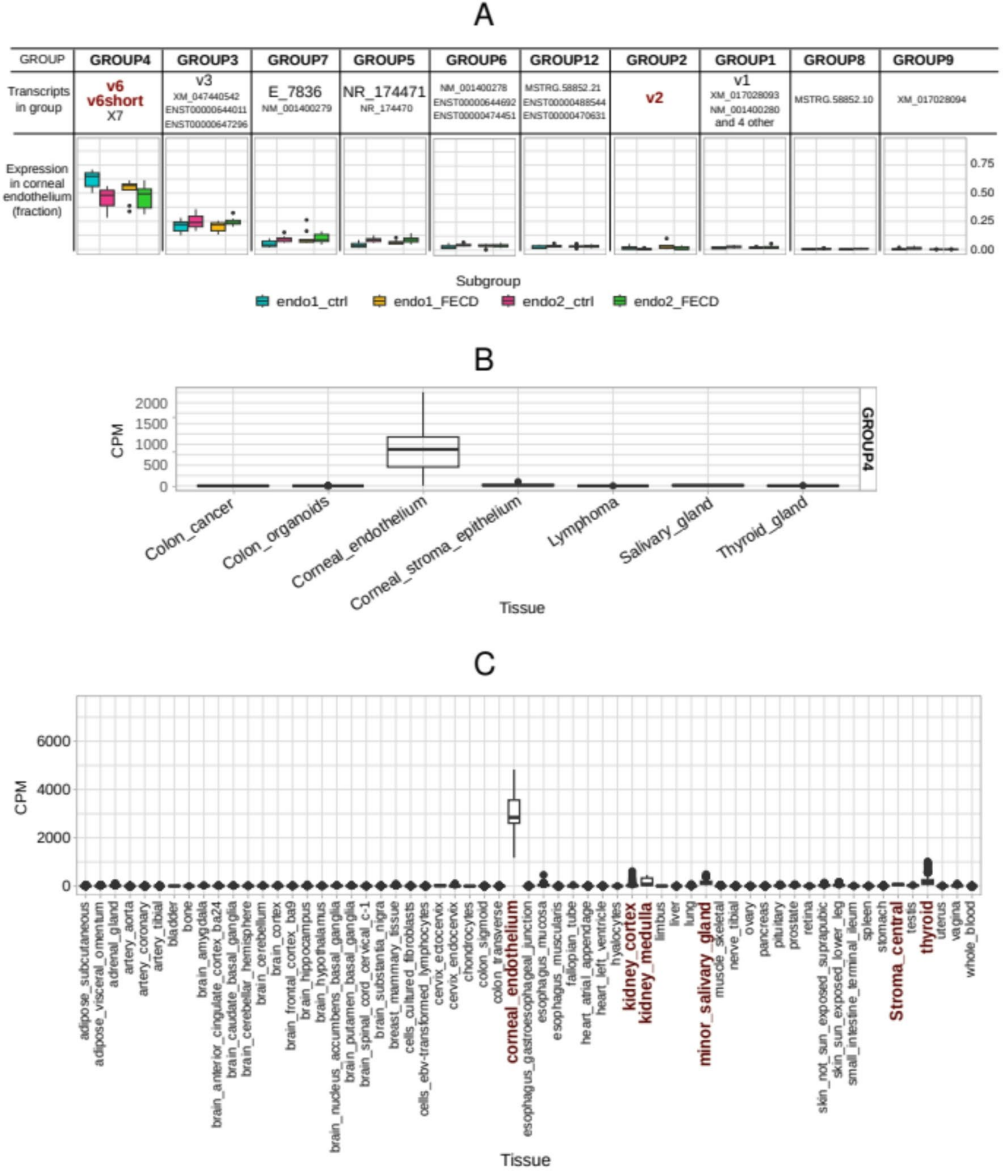
*SLC4A11* transcript groups and overall gene expression. According to the structure of the 5’ end, the *SLC4A11* transcripts were classified into groups. (**A**) Fractions of the *SLC4A11* transcript groups from the total gene expression data in corneal endothelium. Groups of transcripts with maximal fractions of less than 0.1 of total expression among all samples are not shown. Transcript v1 and v2, as well as the top 5 most highly expressed transcripts in corneal endothelium, are marked in red. The following subgroups of samples are shown: endo1_ctrl, control group of the endo1 set; endo1_FECD, FECD group of the endo1 set; endo2_ctrl, control group of the endo2 set; endo2_FECD, FECD group of the endo2 set. (**B**) Expression of *SLC4A11* transcript group 4 (v6, v6short and X7) in corneal endothelium and other datasets. (**C**) *SLC4A11* expression among GTEx samples and two additional sets: corneal endothelium and corneal stroma. Corneal endothelium RNA-seq data from the endo1 and endo2 datasets were combined and analyzed alongside GTEx multi-tissue data. Tissues used for differential expression analysis are marked in red.

The primary distinction between the coding and non-coding full-length *SLC4A11* transcripts described in the literature resides in the 5′-end region. The diversity of 5′ ends among *SLC4A11* transcript variants arises from alternative promoter usage and differential splicing. To delineate the major transcription initiation sites and splicing events within the 5′ region of *SLC4A11*, we grouped the transcripts according to the structure of their 5′ ends, extending up to the exon where the protein-coding isoforms converge to share the common reading frame (Supplementary Figure S2). Figure 5A, Supplementary Figures S4 and S5 shows that a group of three transcripts sharing the same 5’ end as v6 (group 4) is preferentially expressed in the corneal endothelium. This is followed by a group of four transcripts, including v3 (group 3), and then two groups containing E_7836 (group 7) and NR_174471, which are among the five most abundant transcripts. However, the last two groups cannot be considered to encode the SLC4A11 protein. Group 3 is one of the most represented groups in all analyzed datasets (Supplementary Figure S4). Among the two sets of transcriptomes (col_org2 and lymph), group 4 had markedly lower expression levels than did group 3.

Group 4 exhibits substantially higher expression in corneal endothelium compared to other datasets (Fig. 5B; Supplementary Figures S4, S5). Supplementary Figure S5 compares expression of genes with varying tissue specificity across datasets. Group 4 exhibits markedly higher expression in its target tissue (corneal endothelium) compared to other tissues, consistent with the pattern observed for highly tissue-specific genes (e.g., *LYZ*, *TPO*). In contrast, broadly expressed genes such as *PSMB2* and *GPI* show no comparable tissue-specific enrichment within this dataset.

The pronounced expression differences of the primary *SLC4A11* transcript group between the corneal endothelium and other tissues, together with the association of *SLC4A11* variants with hereditary corneal endothelial disorders - but not with thyroid or salivary gland diseases - suggest a highly corneal endothelium–specific expression pattern of *SLC4A11*. We reanalyzed RNA-seq data from the combined control group of the endo1 and endo2 datasets, alongside GTEx multi-tissue data, using a GTEx protocol to unify data processing and reduce technical bias between datasets. This revealed significantly higher *SLC4A11* expression in corneal endothelium samples (Fig. 5C; Supplementary Figure S6), than in any other analyzed tissue, including salivary gland, thyroid, kidney cortex, kidney medulla, or corneal stroma (log2 fold change is 4.34, 4.21, 6.11, 4.21 and 5.24, respectively; p-value < 0.00001) These findings align with our observations of Group 4 (including v6) expression specificity in corneal endothelium and the predominance of hereditary corneal pathologies among carriers of rare *SLC4A11* variants.​

### 5’ RACE analysis of corneal endothelial samples

**Fig. 6:**
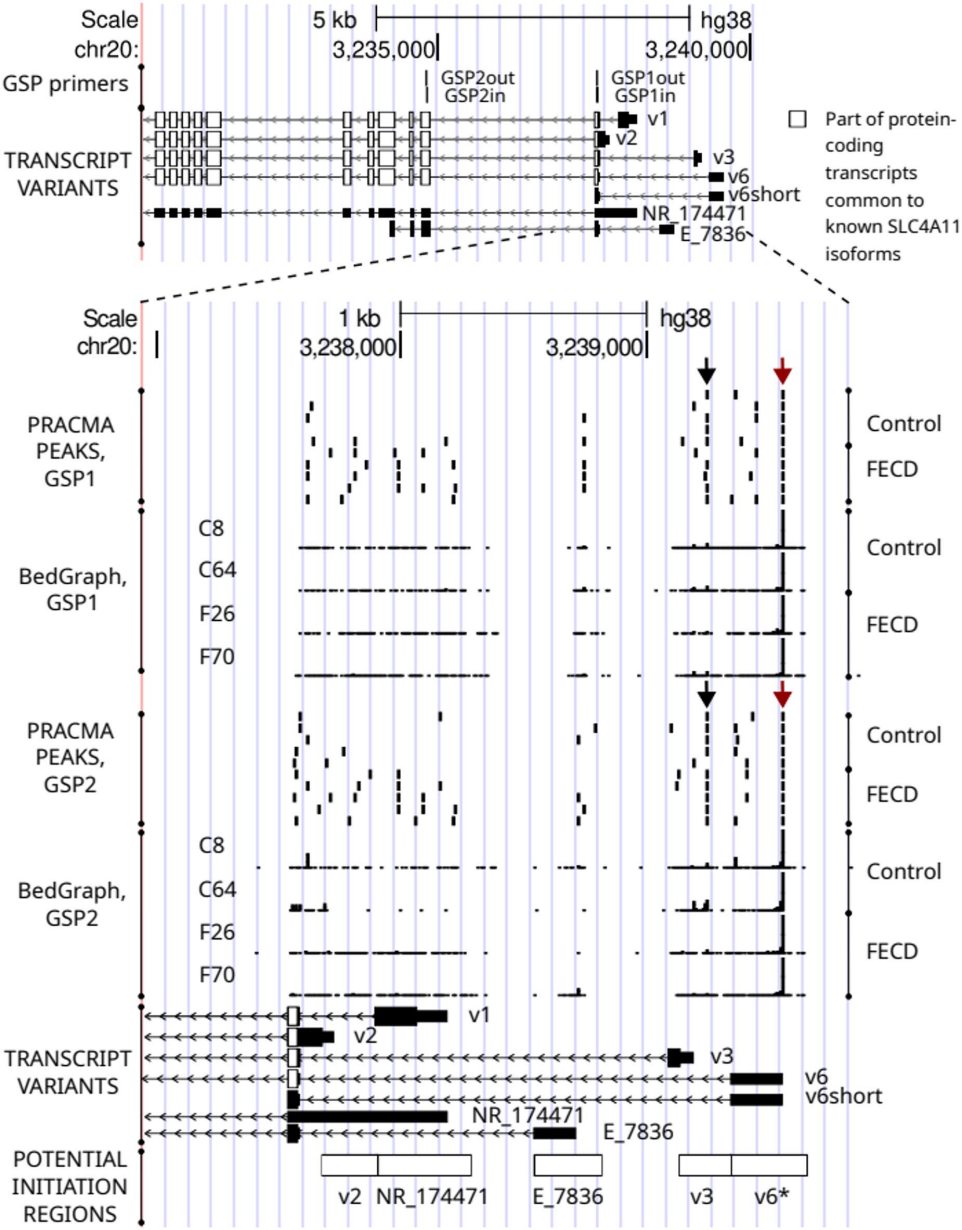
Genomic map with the results of 5’ RACE analysis. The locations of gene-specific primers are shown on the top map. Outward (out) and inward (in) primers bearing the same GSP number were used for different rounds of nested PCR. The locations of the peaks obtained by the pracma package are given for all the samples on the PRACMA PEAKS TRACKs. For two samples from the control group (C8 and C64) and two samples from the FECD group (F26 and F70), tracks with 5’-end transcript coverage in BedGraph format (BedGraph GSP1 and BedGraph GSP2) are also shown. The height of the BedGraph tracks is equal to half of the maximum coverage, which means that the coverage of the first nucleotide of isoform 6 is only visible up to the midpoint. The red arrow shows the location of the peaks that overlap in all samples (the beginning of v6). The black arrow shows the location of the peaks that overlap in most samples (near the beginning of v3). v6* means that the initiation region also corresponds to v6short and GSP1 can detect this transcript.

To characterize the 5’ regions of *SLC4A11* transcripts, localize the main TSSs in the corneal endothelium and validate the transcriptomic findings, 5’ RACE was performed on RNA samples from five healthy donors and five FECD patients. On the PCA plots (Fig. 2A and B), we observed significant heterogeneity of samples without expansion of the CTG18.1 repeats in the *TCF4* gene. Meanwhile, FECD without expansion is much less represented in the population. Therefore, we anticipated better convergence and more unambiguous results when analyzing samples carrying the expansion, and used only this sample group. Two sets of nested gene-specific primers (GSPs) were utilized for 5’ends amplification: GSP1 and GSP2 (Fig. 6). The GSP2 set excluded amplification of the v6short. The coverage of the genome by the 5’ ends of the transcripts was analyzed. The most prevalent TSS across all the samples was identified at chr20:3239559, corresponding to the start of v6 (Fig. 6). To identify other major TSSs, we detected peaks (signal spikes) in the genome coverage. A consistent peak at chr20:3239247 was observed in at least 8 out of 10 samples for both GSP variants, situated 57 bp upstream of the annotated v3 TSS in Ensembl/RefSeq. In some samples, additional peaks were also detected closer to the canonical start of v3, suggesting a possibly dispersed structure of the transcript 3 initiation region.

**Fig. 7:**
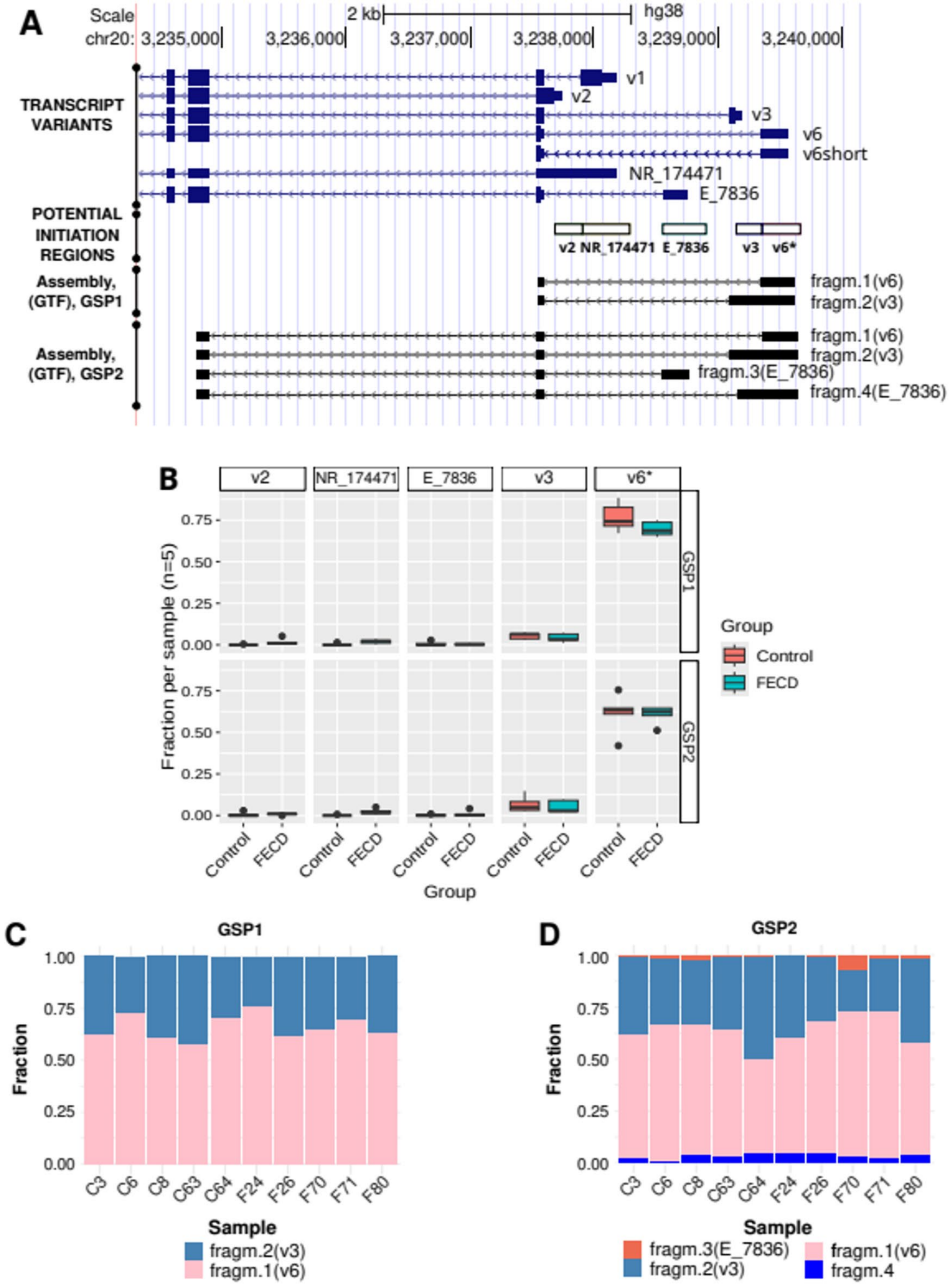
Transcription initiation activity of the genome regions containing start sites of the most abundant transcript variants, and exonic structure of 5’-end sequences. (**A**) Location of potential initiator regions for the most presented transcripts (v2, NR_174471, E_7836, v3, and v6), and exonic sequences of 5’-end fragments de novo assembled using StringTie. v6* means that the initiation region also corresponds to v6short and GSP1 can detect this transcript. (**B**) Fractions of the 5’ ends of transcripts located in the potential initiation regions of v2 NR_174471, E_7836, v3, and v6. (**C**, **D**) Fractions of assembled fragments in samples obtained with GSP1 and GSP2, respectively.

On the basis of these observations and the considerable distances between the TSSs of different transcripts, we hypothesized that *SLC4A11* utilizes multiple promoters, which may be either focused or dispersed. Four potential initiation regions (Fig. 7A) were defined for v2, v3, v6 (and v6short), E_7836 and NR_174471, with lengths in the range of 209–395 bp. These regions were delineated on the basis of reading frame starts, exon boundaries, and the absence of additional ATG codons with strong Kozak sequences. The fractions of the 5’ ends of the transcripts located in the potential initiation regions and the distributions of the peaks among the potential initiation regions are shown in Fig. 7B and Supplementary Figure S7.

No statistically significant differences were found in the fractions of the 5’ ends detected in almost all the initiation regions analyzed between the control and FECD groups (Wilcoxon nonpaired, two-tailed test, significance level for testing: 0.01; Supplementary Table S4). Statistical analysis revealed that TSSs in the initiation region of the noncoding transcript NR_174471 were more frequently detected by GSP2 in FECD patient samples (*p* = 0.0079). The initiation region of v6 was represented in each sample at a significantly higher level than any other analyzed initiation region (chi-square test, significance level set at 0.01; see Supplementary Table S5). The calculated significance levels were no greater than 5.96E-35. Statistically significant differences in the 5’-end fractions were observed between the initiation region of transcript v6 and all other initiation regions within the sample groups obtained with GSP1 and GSP2 (Wilcoxon test, significance level for testing 0.01; Supplementary Tables S6 and S7).

StringTie analysis of the sequencing results (Fig. 7A, C and D; Supplementary Figure S8) unexpectedly revealed a transcript corresponding to v3 with an elongated 5’ UTR, which shares the same TSS as v6 (chr20:3239559). The absence of intervening AUG codons suggests that this transcript encodes the same protein isoform as the canonical variant 3. Therefore, we obtained direct evidence that the main initiation region in the corneal endothelium is focused and produces two predominant transcripts: v6 and v3, with an elongated 5’ UTR. In samples amplified with GSP1 primers, v3 with the long 5’ UTR was approximately twice as abundant as the canonical v3 (0.23 and 0.10 of total detected expression, respectively, Supplementary Table S8). In contrast, GSP2 primers revealed approximately equal fractions of both variants (v3Long: 0.18; v3 canonical: 0.14). We consider GSP2 results more reliable for assessing *SLC4A11* transcripts containing the ORF, as GSP2 amplifies longer 5’-terminal regions and excludes contributions from truncated transcripts potentially arising in the proximal 5’ gene region. Thus, the proportion of *SLC4A11* transcripts derived from the dispersed initiation region of canonical v3 can be estimated as 0.14.​.

The median proportion of 5’-end fragments corresponding to transcripts v6 and v3 out of the total 5’-end fragments was approximately 0.63 and 0.34, respectively (values for GSP2; similar values were observed for GSP1, Supplementary Table S8). Thus, we can estimate the contribution of transcripts v6 and v3 to the overall expression of *SLC4A11*. In doing so, we disregard the minor transcript X7, which shares the same 5’-end as v6, as well as three minor transcripts that share 5’-ends with v3.

The comparison of the read fractions corresponding to transcript v6 between GSP1 and GSP2 revealed no significant difference (Wilcoxon test, *p* = 0.43; Supplementary Table S8), with a median ratio of 1.02. A similar median ratio of 1.06 was observed for transcript v3, which was also not significant (*p* = 1.00). This finding suggests that the substantial expression of v6short estimated by Salmon may be an artifact due to its complete incorporation within v6. Therefore, we conclude that v6 is likely the major contributor to the expression of group 4 transcripts in the corneal endothelium.

The main detected TSS was located within the binding sites of the TATA-box-binding factors TBP and TAF1 in H1-hESC cells according to the UCSC genome browser^35^(Track: Transcription Factor ChIP-seq Peaks (340 factors in 129 cell types) from ENCODE 3). This finding indicates that the TSS is being the initiator with the nearby TATA-box.

Thus, 5’RACE results largely align with transcriptomic analysis, confirming that v3 and v6 are the predominant *SLC4A11* transcripts. Additionally, 5’RACE complements the transcriptomic data by characterizing transcription initiation in the locus, demonstrating v6 predominance over v3, and identifying transcript v3 with an extended 5’ UTR.

### The full-length transcript v6 is present in corneal endothelial cells and encodes v2M36

The v2M36 SLC4A11 protein isoform predominates in corneal endothelium, as shown indirectly by Western blot analysis of lysates^17^. This isoform is thought to arise from translation of transcript v2; however, our data show v2 is not the most abundant *SLC4A11* transcript in corneal endothelium. Moreover, v2 contains an out-of-frame ATG codon (NM_032034, 107 nt) between its first ATG and the v2M36 start codon (same reading frame), which is unusual since upstream out-of-frame ATGs typically reduce main ORF translation efficiency^36–38^.

According to RefSeq data and TransDecoder identification, transcript v6 contains a single ORF starting at its first ATG (with a strong Kozak sequence^17^ that also encodes v2M36 isoform.

The most common translation start site in eukaryotes is the first ATG within a strong Kozak context^39,40^; however, numerous exceptions exist where translation initiates upstream or downstream of this site^41^. Therefore, direct experimental confirmation of transcript v6’s ability to produce v2M36 is crucial.

**Fig. 8:**
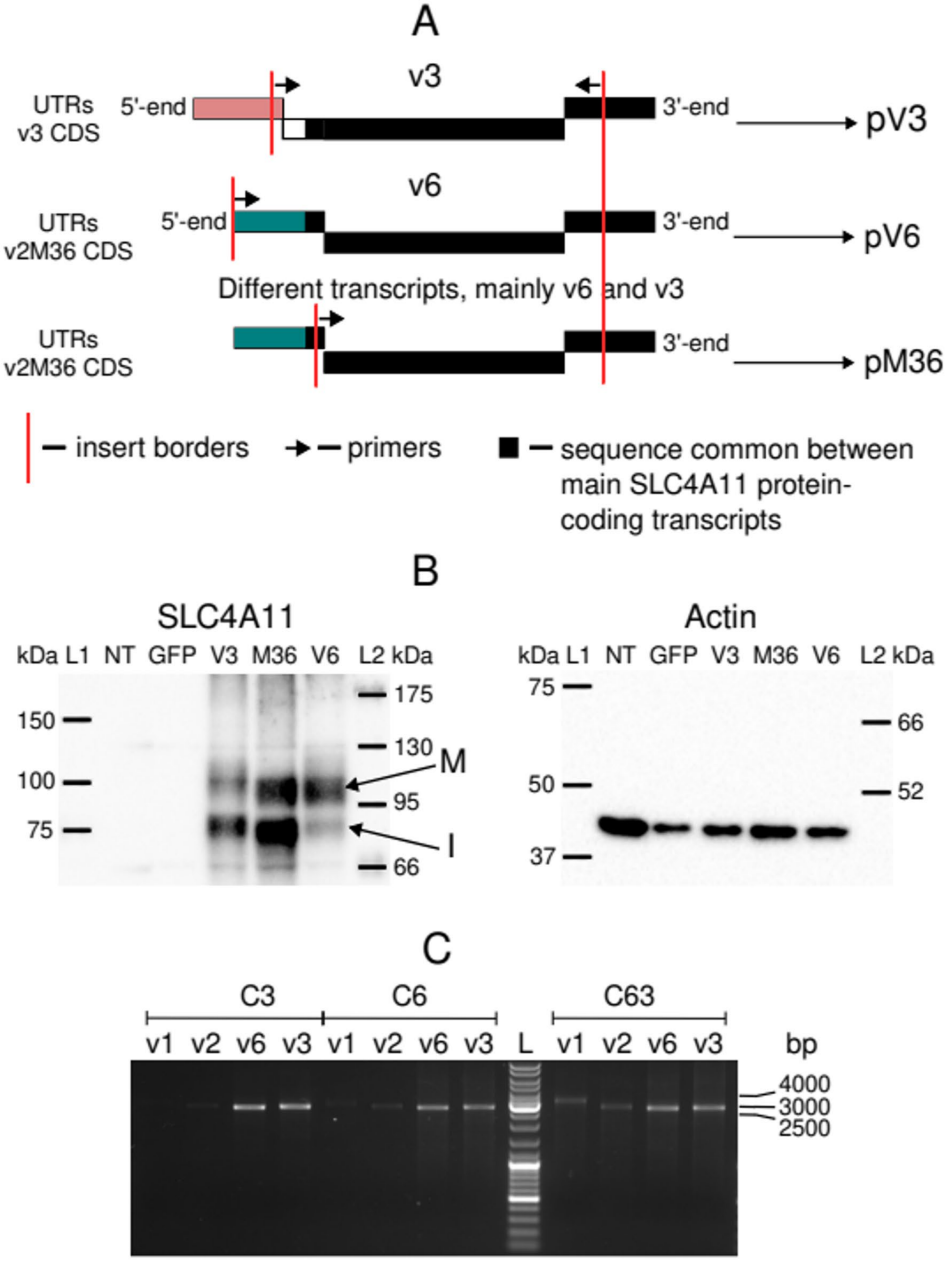
Analysis of the protein-coding potential of transcript v6. (**A**) Generation of plasmid constructs pV3, pM36 and pV6. Boundaries of transcript regions corresponding to the DNA fragment inserted into the plasmid construct are shown. (**B**) Western blot analysis of cell lysates from HEK293 cells transfected with plasmids pV3, pM36 and pV6. The left panel shows immunostaining of the membrane with antibodies against SLC4A11, right panel - with antibodies against beta-actin. L1, L2 – two different protein ladders (see Materials and Methods); NT – no transfection; GFP – transfection with the same plasmid vector as pV3, pVM36 and pV6 with insertion of copGFP gene; v3, M36, v6 – transfection with plasmids pV3, pM36, and pV6, respectively. M – mature protein; I – immature protein. C. Detection of full-length transcript v6 in different samples of corneal endothelial RNA (C3, C6, C63). L – ladder; v1, v2, v3, v6 – transcripts amplified using specific primers.

To validate the predicted coding potential of transcript v6 for v2M36, we generated constructs containing: 1) the v2M36 ORF without the 5’ UTR (pM36), 2) the majority of transcript v6 including the full-length 5’ UTR and v2M36 ORF (pV6), and 3) the v3 ORF (pV3) (Fig. 8A).

Previous studies have shown that various SLC4A11 isoforms (v2, v3, v2M36) display two bands on immunoblots, corresponding to immature (lower band) and mature glycosylated (upper band) forms of the protein^4,15–17^. Western blot analysis revealed two bands of expected sizes in lysates from cells transfected with pM36 and pV3 constructs (Fig. 8B; Supplementary Figure S9). Similarly, transfection with pV6 also produced two bands matching those in the pM36 sample, demonstrating that the ORF encoded by transcript v6 translates into v2M36. For cloning transcript fragments into constructs, amplification was performed using cDNA from corneal endothelium. Notably, for the pV6 construct, nearly the entire transcript v6—including all exon-exon junctions, the full-length 5’-UTR, ORF, and part of the 3’ UTR—was amplified, providing evidence for the presence of the full-length transcript v6 in corneal endothelium (Fig. 8A).

Further evidence for full-length transcript v6 in other endothelial samples was obtained using primers amplifying the v6 fragment encompassing the coding sequence (CDS) and all exon-exon junctions. For comparison, transcripts v3, v1, and v2 were also amplified. While it was not possible to design absolutely specific primers for v6, v3, v1, or v2 targeting fragments with the complete CDS and all exon-exon junctions, v3 and v6 primers amplified only target transcripts among the top five most highly expressed in corneal endothelium (v6 primers can also amplify the proposed transcript X7), whereas v1 and v2 primers also amplified NR_174471.​

As shown in Fig. 8C, amplification of expected-size fragments with v6 (2697 bp) and v3 (2744 bp) primers in three samples supports the presence of full-length v6 and v3 transcripts in corneal endothelium. Weaker amplification with v2/NR_174471 primers (2736 bp; identical expected size for both transcripts) was observed in all samples. Amplification with v1/NR_174471 primers was also detected, but in sample C63, the amplicon migrated slightly slower than in C6, suggesting predominant amplification from v1 (expected 3117 bp) in C6 and from NR_174471 (expected 2863 bp) in C63.

## Discussion

In this work we characterized the expression of *SLC4A11* in corneal endothelium to resolve some discrepancies existing in literature. Using transcriptomic data analysis and 5’RACE we demonstrated that predominant protein-coding transcript variants in corneal endothelium are v6 and v3 (presented in corneal endothelium as canonical transcript and transcript with long 5’UTR). It was shown high specificity of *SLC4A11* gene and transcripts expression for corneal endothelium. Also some features of promoter region functioning were described: location of main TSS and focused transcription initiation.

To date, many studies on *SLC4A11* gene expression and its protein functions in the corneal endothelium have been predicated based on the existence of three major transcripts (v1, v2, and v3) with different 5’ ends and N-terminal fragments of their protein products. Before 2015, all research on *SLC4A11* was based on the assumption that the main transcript corresponds to v2, with protein translation initiating from the first ATG codon of this variant.Kao et al. first analyzed multiple isoforms (v1, v2 and v3) with specific RefSeq identifiers, indicating that these isoforms were catalogued in the NCBI RefSeq database^15^. From our perspective, the two publications by Kao et al. (2015^15^, 2016^18^) and the study by Malhotra et al. (2019)^17^ currently represent the main investigations into the relative abundance of *SLC4A11* transcript variants and protein isoforms. In these studies SLC4A11 transcripts 1–3 expression was analysed by qPCR using donor corneas, without indication that any samples were obtained from individuals with FECD. From 2015 until recently, all publications we identified (36 studies containing information on human *SLC4A11* transcript variants or protein isoforms within references 1–79 of Supplementary File 1) referred to no more than three transcript variants of *SLC4A11*. Transcript v6 gained validated RefSeq status (NM prefix) in 2022 (previously predicted XM_011529384), remaining untested in key 2015–2019 studies. Prior analysis of 5’-terminal sequences of *SLC4A11* transcripts in corneal endothelium was also lacking.

Correct isoform selection is critical for investigating protein localization and function. Studies have demonstrated that subtle variations in the N-terminal regions—differentiating isoform 1 from isoforms 2 and 3—can dictate localization: isoform 1 resides in the cytoplasm, unlike the membrane-associated isoforms 2 and 3 ^18^. Our data indicate that transcript v6 is the predominant *SLC4A11* transcript (vs. low expressed v2) in the corneal endothelium. Also we obtained evidence that whole size transcript v6 present in corneal endothelium and able to be translated into v2M36. In the study by Malhotra et al.^17^, direct Western blot analysis of corneal endothelium lysates demonstrated that v2M36 is the predominant form of the SLC4A11 protein in the corneal endothelium. Our data support v2M36 as the main *SLC4A11* isoform in corneal endothelium and reinforce the rationale for prioritizing v2M36 over v2M1 in functional studies.

Other transcripts encoding v2M36, such as XM_017028094, may also be expressed in some tissues (e.g., the colon). This phenomenon should be considered in future analyses of the expression of this gene in different tissues and cell types, particularly when investigating its role in the development of glutamine-addicted cancers.

Identification of the principal transcript and corresponding protein isoform is essential for accurately determining the position of potentially pathogenic variants within the protein-coding sequence of the gene. Certain rare variants located in the 5′ untranslated region of *SLC4A11* may therefore warrant re-evaluation in light of transcript-specific differences. Most previous studies investigating rare nucleotide variants in *SLC4A11* associated with FECD and CHED have focused on sequencing genomic regions encoding the protein translated from the first ATG of transcript v2 (NP_114423). Notably, several reports have described variants leading to amino acid substitutions up to the thirty-sixth methionine, which corresponds to the first residue of the v2M36 isoform. For example, Vithana et al. demonstrated an association between FECD and a deletion, NC_000020.11:g.3237582_3237583del, in transcript v2 (NM_032034.4:c.99_100del)^4^. Although this deletion was expected to cause a shift in the reading frame of the protein, in light of new data, its effect may be related to disruption in the structure of noncoding functional elements (transcription regulation elements, splice branch point site etc) rather than changes in the coding sequence. In light of these findings, the structure of sequences corresponding to splice sites in the 5’ UTRs of transcripts v6 and v3 has become increasingly important for ophthalmic genetics. Correct splicing of these transcripts is essential for proper *SLC4A11* translation and function.

Thus, the predominance of v6 expression in corneal endothelium, with its protein product matching the main protein isoform v2M36 identified in corneal endothelium, indicates that this transcript should be prioritized for annotating rare *SLC4A11* variants henceforth. Changes in the *SLC4A11* nucleotide sequence should be interpreted relative to the reading frame and splice sites of this transcript. Our data raise the question of designating v6 as a clinically significant SLC4A11 isoform, such as MANE (Matched Annotation from NCBI and EBI) Select or MANE Select Plus.

Currently many studies on *SLC4A11* gene expression were limited to the thyroid gland, salivary gland and tumors due the limited access to the corneal tissues. However, the information regarding the association of *SLC4A11* with FECD, CHED and Harboyan syndrome prompted us to address the issue of tissue-specific expression of the *SLC4A11* gene. Current our analysis and by Chu et al.^26^ clearly demonstrated that overall transcriptomic profile of the endothelium differs significantly between samples with and without FECD (Supplementary Figure S1), but these differences are not explained by, or do not correlate with the expression of different *SLC4A11* transcript-variants (Fig. 2). However, the high expression of *SLC4A11* and its transcripts v6 and v3 is strongly correlated with the corneal endothelium, irrespective of pathology, compared to all other tissues (Figs. 3A, 4 and 5, Supplementary Figure S4). Some FECD samples without repeat expansion in the *TCF4* gene (F9, F10, and F23) presented differences in the expression of *SLC4A11* transcripts: these samples did not cluster with other corneal endothelial samples in the PCA plots. This may be related to FECD not being associated with *TCF4* repeat expansion or indicate a loss of *SLC4A11* gene or transcript expression in severely compromised corneal endothelium with advanced FECD. However, at this point, we can only speculate about the association between FECD and the expression of *SLC4A11* transcripts in these patients.

Due to differences in tissue complexity, the observed expression level of a gene or transcript in a given tissue may reflect not only its intrinsic expression in that tissue but also the proportion of cells that exhibit high versus low expression. Nevertheless, a higher abundance of the gene product in a specific tissue suggests functional importance there, particularly when that tissue is compared with a broad range of others. Rare *SLC4A11* variants are associated with inherited corneal endothelium diseases, but not with pathologies of tissues previously reported to highly express *SLC4A11* (thyroid, salivary glands, kidney). This aligns well with the significantly elevated *SLC4A11* expression in corneal endothelium compared to these or any other tissues.

The data regarding the location of the *SLC4A11* promoter and its initiator region have also been inconsistent. The initial article on *SLC4A11*^14^, which led to the assumption that v2 was the major transcript in the corneal endothelium, stated that a putative GC box included in the promoter is located immediately upstream of the transcript v2 start site, but no CCAAT or TATA boxes are present. Subsequently, bioinformatic analysis identified a promoter ~ 1 kbp upstream of the v2 transcription start site^22,23^, with demonstrated activity via luciferase assay^23^. This region does not overlap with transcript 2’s transcription initiation site. These findings are consistent with the preferential initiation of transcription at the start site of v6, as it is part of this promoter region. Conventional luciferase reporter assays, used to demonstrate the activity of the promoter region, provide important data on promoter function and regulation. Nevertheless, demonstrating promoter activity of a genomic region alone does not confirm its function as a promoter in a specific tissue, since promoter activity is largely regulated by chromatin composition at the locus and DNA methylation^43,44^. Therefore, only direct identification of transcription initiation through TSS mapping provides definitive evidence of promoter activity in a given tissue. Our results thus offer the first direct confirmation that this region functions as the principal promoter of *SLC4A11*, driving transcription initiation in the corneal endothelium. The binding of TBP and TAF1 with the start site of v6 also points to this TSS being the initiator with the nearby TATA-box. Interestingly, the active initiation of transcription predominantly from a single point (observed through 5’ RACE) is characteristic of promoters that typically contain key regulatory elements near their transcription start sites (TSSs) and are highly active in terminally differentiated cells within adult tissues^45^. This observation aligns with the significantly higher median expression levels of *SLC4A11* and its transcripts 3 and 6 in the endothelial dataset samples than in the other datasets.

Data related to gene expression regulation are crucial for studying the molecular mechanisms of pathogenesis, developing therapies, and diagnosing associated pathologies.

A more comprehensive understanding of the main *SLC4A11* transcript variants and promoter region functioning will facilitate better insight into how the expression of this gene is regulated at the transcriptional level. *SLC4A11* expression in corneal endothelium increases under oxidative stress^23^. The promoter activity of the DNA fragment containing the TSSs of transcripts 6 and 3 is also enhanced under these conditions. However, to better understand SLC4A11’s role in oxidative stress, it is important to determine which specific transcripts are upregulated in corneal endothelial cells—including the two main protein-coding transcripts (v6 and v3), as well as E_7836 and the noncoding RNA NR_174471. The localization of the primary initiator may be crucial for identifying additional regulatory elements in the locus, such as transcription activators within the promoter region, enhancers, and silencers.

Furthermore, understanding the regulation of *SLC4A11* may be valuable for advancing cell-based therapies for CHED and FECD. Cultured cell transplantation has been considered as a treatment for endothelial dystrophies. However, it has been shown that during mature corneal endothelium cell culture, the expression of many genes important for corneal endothelial function, including *SLC4A11*, is suppressed at early passages^46^. Specifically, *SLC4A11* expression is almost completely lost after passage 2 of endothelial cell culture^47^, although this protein appears necessary for normal endothelial function. High expression of the proper major *SLC4A11* isoforms may serve as a marker of functional competence in transplanted cells when selecting therapeutic approaches.

Regarding the search for potential pathogenic variants within the regulatory regions of *SLC4A11*, three studies have screened the promoter region encompassing the TSS of v6 for CHED-associated mutations across a total of 22 families, but no causal variants were identified^22,48,49^. The recessive inheritance pattern of CHED implies that disease manifestation requires near-complete loss of SLC4A11 function. The functionality of promoter and enhancer elements is largely determined by their correspondence to consensus sequences^50^. Single-nucleotide substitutions at most positions within a promoter/enhancer element are generally insufficient to completely abolish its activity. Therefore, the development of CHED is likely caused only by more extended homozygous disruptions of regulatory elements, such as deletions or insertions of multiple nucleotides. This may explain the current absence of identified pathogenic promoter variants despite comprehensive screening. We propose that pathogenic variants in the regulatory sequences of *SLC4A11* associated with CHED may exist, but are likely to be very rare. No analogous studies have investigated *SLC4A11* promoter variants in FECD. Given that part of FECD cases lack established genetic markers, further analysis of *SLC4A11* regulatory elements may elucidate pathogenesis in some unresolved cases.

The main limitations of this study relate to potential artifacts of the methods employed. A common problem with most expression analysis methods based on short-read sequencing is the assessment of expression of transcripts when one is nested within another due to the lack of uniqueness. Also, a separate problem is the assessment of the expression of short transcripts due to the high uncertainty of measuring the expression level by fragments comparable to the length of the transcripts.But a single-molecule whole-transcriptome sequencing as provided by Oxford Nanopore is almost impossible for the real-world tissue samples especially for the such unique and small as corneal endothelium. While comparisons across datasets from different sources cannot completely eliminate technical bias, the bias minimization achieved here suffices to demonstrate the high tissue specificity observed for genes such as *TPO*, *CD19*, *LYZ*, *KERA*, and *SLC4A11*. Factors like FECD stage and sample quality may also influence corneal endothelium RNA analysis. Nevertheless, the high consistency between results from diverse datasets and methods supports the validity of our main conclusions.

## Materials and methods

### Tissue specimen collection

Patients with FECD or keratoconus who underwent corneal transplantation at the S. Fyodorov Eye Microsurgery Complex Federal State Institution provided consent to preserve their corneal samples. Patient diagnosis and staging were conducted as described by Skorodumova et al.^51,52^. Donor corneal samples were obtained from the Eye Bank of the S. Fyodorov Eye Microsurgery Complex Federal State Institution. Corneal endothelium samples from patients with FECD and donors were collected as described by Nikitina et al.^24^. Blood was also collected from FECD patients, and iris or sclera from donors, for DNA extraction and CTG18.1 genotyping as described in Nikitina et al. Patients with keratoconus underwent deep anterior lamellar keratoplasty (DALK). The samples from the four keratoconic patients included three corneal layers: the corneal epithelium, Bowman’s membrane, and stroma. The samples from the other six keratoconic patients (after epithelial debridement) included only Bowman’s membrane and stroma, as indicated in Supplementary Files 2 and 5. The donor corneal samples always underwent epithelial debridement at the S. Fyodorov Eye Eight donor corneal samples included only Bowman’s membrane and stroma. Both keratoconic and donor corneal samples were divided into central (semicircle with a 2.5 mm diameter) and peripheral parts for comparative analysis.

Colorectal tumor fragments were collected from the Sechenov University Biobank in transport medium and subsequently transferred to the laboratory. Small fragments (3–5 mm) were separated into IntactRNA (Evrogen, Russia) for long-term storage at -80 °C.

### Characteristics of patients and samples

The characteristics of the FECD patients and the samples labeled F1–F19, along with donor samples C1, C3, and C5–C8, are provided in Nikitina et al.^24^ and also in Supplementary Files 2 and 5. CTG18.1 genotyping was performed for all FECD patients and donors using a combination of short tandem repeat and triplet-primed PCR analyses, as described in Skorodumova et al. The characteristics of corneal tissue fragments (which included stroma) from patients with keratoconus and donors are provided in Supplementary Files 2 and 5 (dataset kc).

### RNA isolation

Total RNA isolation from tissue samples varied on the basis of the disruption and homogenization methods employed. The downstream steps of RNA isolation were largely consistent across all the samples. Corneal endothelium samples from FECD patients and donors were disrupted and homogenized using the TissueRuptor II (Qiagen, Germany) for 20 seconds. Keratoconic, donor corneal, and colorectal tumor samples were cut into small pieces with a scalpel, followed by homogenization via a TissueLyser (Qiagen, Germany) with 4.75 mm diameter stainless steel beads for 30 seconds at maximum speed. Total RNA was isolated from all samples using an RNeasy Mini or RNeasy Micro Kit (Qiagen, Germany) according to the manufacturer’s protocol. DNA traces were removed using a TURBO DNA-free Kit (Thermo Fisher Scientific, Waltham, Massachusetts, USA). The RNA integrity number (RIN) was assessed via a Bioanalyzer 2100 instrument (Agilent, USA) with an Agilent RNA Pico 6000 Kit (Agilent, USA) or via TapeStation 4150 (Agilent, USA) with High Sensitivity RNA ScreenTape assay (Agilent, USA) RIN values were obtained for 83% of the endothelial RNA samples prepared in this study (median RIN: 6.6), with only two samples below 6 (5.0 and 5.6). All samples used for 5’ RACE had RIN ≥ 6.5 (median RIN = 6.9, Supplementary File 5).

### 5’ RACE

The 5’ RACE protocol was adopted from the first RACE protocol incorporating PCR suppression^53^ and a protocol for amplifying the 5’ ends of T-cell receptor mRNAs^54^. The obtained protocol included reverse transcription with template switching, followed by two-step nested PCR with PCR suppression. Full-length first-strand cDNAs with adaptor sequences at the 5’ end were synthesized via the use of 25–100 ng of total RNA with the SMARTer RACE 5’/3’ Kit (Takara, Japan) following the manufacturer’s protocol, excluding the use of a 1 µM template switch oligonucleotide (TSO) with a 17 bp unique molecular identifier (UMI) instead of the SMARTer II A oligonucleotide. The TSO consisted (from the 5’ end to the 3’ end) of a standard sequence for annealing the long step-out primer (23 nt), UMI (17 nt), a service sequence (5 nt) to separate the UMI and riboG-tail necessary for correct processing of NGS reads, and a riboG-tail (5 nt) for template switching. UMIs enable the removal of PCR duplicates, facilitating the comparison of DNA fragment counts^55^. The riboG tail is comprised of five guanine bases in ribonucleosides, with the last riboguanosine featuring an LNA modification employed in certain template switching-based protocols^56,57^ to increase the annealing of the TSO. The SMARTer RACE 5’/3’ Kit employs an oligo-dT reverse transcription primer to enrich for protein-coding transcripts. PCR amplification was conducted in two rounds: twelve cycles with a melting temperature of 62 °C and 29–31 cycles with a melting temperature of 65 °C. The outer gene-specific primer (GSP) and a commonly used long step-out primer from the SMARTer RACE 5’/3’ Kit (Takara), with a 3’ end corresponding to the 23 bp TSO sequence, were utilized for the first round. The inner GSP and a commonly used short library primer from the SMARTer RACE 5’/3’ Kit (Takara, Japan), corresponding to the last 22 bp of the long library primer sequence, were used for the second round. Both rounds of PCR were conducted with the Q5 High-Fidelity 2x Master Mix (New England Biolabs, USA) and primers at a final concentration of 0.25 µM. The subsequent use of the long step-out primer and the short library primer suppressed the amplification of some nonspecific products from the reverse transcription stage^53,54^. Two different combinations of the inner and outer GSP were employed.

The first combination consisted of GSP1out and GSP1in primers annealed at the second exon of transcript v3 (NM_1174089/ENST00000642402 in Ensembl) to detect the four transcripts that were most highly expressed according to transcriptomic dataset analysis (v3, v6, v6short, and NR_174471) as well as transcript v1 and v2, which were identified as the main *SLC4A11* transcripts in the corneal endothelium according to the literature^17,58^. The second combination comprised GSP2out and GSP2in primers annealed at the third exon of transcript v3 to provide a set of 5’-end transcript sequences without isoform v6short. All oligonucleotide sequences are provided in Supplementary Table S1. Equal amounts (in ng) of the amplicons from the same cDNA sample with GSP1 and GSP2 were combined and used for Illumina MiSeq library preparation.

### Library preparation and NGS sequencing

During the preparation of the whole-transcriptome libraries, ribosomal RNA was depleted using a NebNext rRNA Depletion Kit (Human/Mouse/Rat) (New England Biolabs, USA). Transcriptome libraries were constructed using a NEBNext Ultra II Directional Library Prep Kit for Illumina (New England Biolabs, USA) and Multiplex Oligos for Illumina (96 Indices) (New England Biolabs, USA). The resulting paired-end libraries were sequenced on an Illumina HiSeq 2500 instrument with 2 × 125 cycles using a HiSeq SBS Kit v4 (Illumina, USA). The 5’ RACE dual-barcoded libraries were prepared using the NebNext Ultra II DNA Library Prep for Illumina (New England Biolabs, USA) and NEBNext Multiplex Oligos for Illumina (Dual Index Primers Set 1) (New England Biolabs, USA) according to their manuals. Sequencing was conducted on an Illumina MiSeq sequencer using a MS-102-3003 MiSeq Reagent Kit v3 (600 cycles). NGS sequencing was carried out via the core facilities of the Lopukhin FRCC PCM “Genomics, Proteomics, Metabolomics” (http://rcpcm.org/?p=2806).

### Transcriptomic data selection

For the analysis, we used our own transcriptomic datasets and datasets from the GEO portal. The data for the samples and datasets can be found in Supplementary File 2. Samples exhibiting low *SLC4A11* expression (below 0.5 TPM) were excluded from the subsequent analysis.

The endothelial dataset endo1 was obtained in our laboratory. Dataset endo1 consisted of transcriptomes described by Nikitina et al.^24^ and four additional corneal endothelial transcriptomes (from two donors and two patients with FECD, Supplementary File 5) obtained following the same protocol. The dataset endo2 was obtained from the GEO portal (GSE86356)^26^.

The kc dataset obtained in our laboratory comprises the transcriptomes of 18 paired corneal tissue fragments (central and peripheral). According to our preliminary observations, central and peripheral fragments from the same individual presented similar expression levels of the *SLC4A11* gene and its transcripts; therefore, we only analyzed the inner fragments. Additionally, one keratoconic sample (KC_16_in) consisting of stroma and Bowman’s membrane was excluded from the analysis because of low *SLC4A11* expression.

According to the GTEx (the data used for the analyses described in this manuscript were obtained from the GTEx portal on April 30, 2024^59^), the two tissues with the highest median expression of *SLC4A11* are the thyroid and minor salivary gland. Transcriptome datasets corresponding to these tissues were obtained from the GEO portal (GSE16572431, GSE17189632) and named thyr (thyroid gland)^27^ and saliv (salivary gland)^28^. We analyzed ten randomly selected control samples from these datasets.

*SLC4A11* plays a crucial role in the development of glutamine-addicted tumors^34^; therefore, we anticipated its expression in cancer samples. We included tumor samples from two datasets, namely, col_can (colorectal cancer transcriptomes obtained in our laboratory, under other publication) and lymph (Project ID in ENA: PRJEB3031237)^32^, which expressed *SLC4A11* at a level greater than 0.5 TPM. The col_can dataset consisted of 16 colon tumor transcriptomes. Six of them were included in the analysis because they expressed *SLC4A11*, whereas the others are under other publications. The lymph dataset consisted of 10 samples prefiltered earlier (B-cell lymphomas and leukemias from Caucasian men older than 40 years). Four samples expressed *SLC4A11* and were included in the analysis. Additionally, we used normal colon organoids from the col_org1 (5 samples) and col_org2 (12 samples) datasets (GSE16728533 and GSE19616834, respectively)^30,31^.

### Transcriptome annotation file obtainment

To compensate for the incompleteness of the particular transcript databases, we combined all unique *SLC4A11* transcripts from Ensemble and RefSeq. Transcripts with identical exon‒exon junctions and the start and end of the CDS were identified as common for Ensemble and RefSeq. The difference in the length of the 5’ UTR of the matched transcripts was up to 21 nucleotides. The correspondence between the transcript IDs in the Ensembl and RefSeq annotation files and the transcript numbers commonly used in the literature are summarized in Supplementary File 6. The GENCODEv43 GTF file (based on Ensembl annotation and containing 12 unique *SLC4A11* transcripts), was used as the core GTF file and was supplemented with 20 transcripts unique to RefSeq annotation (GRCh38.p14). The resulting GTF file was used for transcriptome-guided assembly of the corneal endothelium transcriptome via the StringTie package^60^. Samples of the dataset endo1 were used for transcriptome assembly with options –fr and specifying the reference annotation through the flag -G. GTF files obtained for all samples were merged by StringTie with options –merge and -i for keeping merged transcripts with retained introns. The StringTie output GTF file was processed by GffCompare (0.12.6)^61^, and all *SLC4A11* transcripts (including 2 novel variants) in the core GENCODEv43 file were replaced by *SLC4A11* transcripts from the output file of GffCompare. The annotations of all 29 *SLC4A11* transcript variants are provided in Supplementary File 3. The TransDecoder package (version 5.5.0) was used to predict ORFs and identify potential coding sequences within the transcriptome data. Annotation of potential reading frames corresponding to novel fragments is provided in Supplementary File 4.

### Transcript and gene expression analysis

An annotation file (GTF) that contained 29 *SLC4A11* transcripts was used to evaluate gene and isoform expression levels. The *SLC4A11* transcript expression was quantified via the Salmon (1.3.0) package^62^. Subsequent analysis was performed in RStudio (3.6.3)^63^. Tximport (1.26.1) was used to integrate the outcomes of the RNA-seq-based gene expression analysis programs following Salmon^64^. The expression of the *SLC4A11* gene and transcripts was subsequently analyzed using the EdgeR package (2.0.7)^65^. For all datasets the data were normalized via the trimmed mean of M-values (TMM) method for non-biological heterogeneity diminishing. The expression levels of each gene and/or transcript of the *SLC4A11* gene were calculated as RPKM. The total expression of selected *SLC4A11* genes and transcripts was visualized using the PCAtools (2.10.0) and ggplot2 (3.5.0) packages^66^. To analyze the underlying trend in the expression of 5’ regions of transcripts, transcripts were grouped on the basis of identical 5’ end fragments (up to the first exon common to the reading frames of known protein isoforms), as shown in Supplementary Figure S2. Three small, possibly noncoding transcripts (MSTRG5852.21, ENST00000488544 and ENST00000470631) located near the end of the gene were grouped together. An analysis of potential differences in the expression of *SLC4A11* transcripts between groups was conducted using the nonpaired two-tailed Wilcoxon test, which was set at a significance level of 0.01, in RStudio software. Differences in transcript expression levels within the same sample group were analyzed using a paired one-tailed Wilcoxon test, with a significance level of 0.01.

For *SLC4A11* expression analysis control samples from corneal endothelium datasets endo1 and endo2 were combined, and processing was performed following the GTEx Consortium RNA-seq pipeline v10 (https://github.com/broadinstitute/gtex-pipeline/tree/master/rnaseq). The data from five donor corneal stroma samples were processed identically. Briefly, reads were aligned to the Broad Institute GRCh38 reference genome excluding ALT, HLA, and Decoy contigs, using STAR (v2.7.10a). PCR duplicates were identified and marked using the duplicate-marking script from the GTEx RNA-seq pipeline with default parameters. Gene expression quantification (in counts) based on GENCODE v39 annotation and quality control were performed using RNA-SeQC (v2.4.2).

Quality control thresholds followed those described in the GTEx v8 publication^67^. Samples were excluded if the total number of mapped reads was < 10 million, the mapping rate < 0.2, the intergenic rate > 0.3, the base mismatch rate > 0.01, or the rRNA rate > 0.3. For paired-end data, samples were also excluded if End 1 or End 2 mismatch rates exceeded 0.02. Twenty-two of 23 endothelial transcriptomes and all 5 corneal stroma transcriptomes passed quality control.

GTEx read counts per each sample were downloaded from GTEX site (https://gtexportal.org/home/downloads/adult-gtex/bulk_tissue_expression)^68^.

The resulting raw count files (combined corneal endothelium dataset, corneal stroma dataset and GTEx data) were then normalized. For each individual file, DEseq2 1.^42^.1^69^ function estimateSizeFactors() with default parameters was used to calculate median-of-ratios size factors for samples. Differential gene expression was calculated using DEseq2 1.^42^.1^69^ function DESeq() with default parameters using a model that included two parameters: sex and tissue type. Nine genes were selected for analysis: *TPO*,* LYZ*,* EPCAM*,* SLC4A11*,* KERA*,* CD19*,* TCF4*,* GPI*, and *PSMB2*. Pairwise comparisons of expression levels were performed between the corneal endothelium and the following tissues: central corneal stoma, thyroid, minor salivary gland, kidney cortex, kidney medulla, sigmoid colon, and transverse colon. For visualization additionally CPM for each sample was calculated by dividing each resulting count value by the sum of all counts for the sample and multiplying by million.

### 5’ RACE data analysis

Adapter sequences (Illumina Universal Adapter) were removed from the reads using the Cutadapt program (4.8). Reads shorter than 93 nucleotides were excluded from further analysis. Additionally, Cutadapt was used for dividing GSP1 and GSP2 read pairs and read orientation unification. The UMI-tools program (1.1.5) was used to eliminate PCR duplicates. Adapter sequences at the 5’ end were removed using the Cutadapt program. The STAR program (2.7.11a) was utilized for mapping reads to a reference genome (GRCh38 assembly). The BAM files were sorted and indexed using the SAMtools software (version 1.19.2). Deduplication was then conducted using UMI-tools to eliminate duplicate reads resulting from PCR amplification. Unpaired reads were eliminated via SAMtools software to correct mating and filter unpaired reads. Consequently, the BAM files were sorted, deduplicated and contained only paired reads. The obtained BAM files were used to create files with coverage of the genomic region with coordinates chr20:3234809–3,259,559 (the first coordinate is the 5’ end of GSP2 in the primer, and the second coordinate is 20 kb upstream of the outermost TSS point of *SLC4A11* transcripts according to RefSeq and Ensembl annotation files) by the start points of the reads. BedGraph files with one nucleotide resolution coverage were obtained. The coverage of each nucleotide was converted into a fraction of all reads at the locus under investigation, and peak finding via the pracma package was performed. The function findpeaks, with parameters minpeakheight = 0.003 and minpeakdistance = 100, was employed. We applied a lenient filter with minpeakheight = 0.003. In some samples, this height was ≤ 1/100 of the tallest peak and corresponded to 1–2 detected 5’-end fragments—unlikely reliable signals but rather transcriptional noise or method artifacts. The minpeakdistance = 100 (100 bp) parameter was selected based on literature estimates of core promoter sizes^45^. We expected this setting to detect no more than one peak per core promoter.

Potential initiation regions were selected on the basis of the genomic context. The potential initiation region of transcript v2 (chr20:3237679–3237894) includes the TSS of NM_032034 and spans from the ORF of v2 to the first ATG upstream of the TSS with a strong Kozak sequence (TIS Predictor, https://www.tispredictor.com/kss)^70^. The potential initiation region for NR_174471 (chr20:3237895–3238290) included the TSS of NR_174471 and spans from ATG with a strong Kozak sequence in the first exon of this transcript to a point 100 bp upstream of the potential start site (maximal size of the dispersed promoter initiation region). Potential initiation regions of transcripts E_7836 and v6 (chr20:3238548–3238813 and chr20:3239348–3239659, respectively) included their TSSs and were located from the border between the first exon and first intron to a point 100 bp upstream of the TSS. The potential initiation region for transcript v3 (chr20:3239138–3239347) includes its TSS and spans from the start of the v3 ORF to the first exon of v6. Fractions of reads falling into each potential initiation region were visualized by ggplot2 (3.5.0)^55^. Statistical significance of differences in the fractions of TSSs detected in all analyzed initiation regions between the control and FECD groups was determined via the nonpaired, two-tailed Wilcoxon test with the significance level set at 0.01 in RStudio software. The v6 initiation region in each sample was compared with the other initiation regions analyzed using the chi-square test with a significance level of 0.01. Additionally, statistically significant differences in TSS fractions were analyzed between the transcript v6 initiation region and all other initiation regions within the sample groups obtained with GSP1 and GSP2. The paired two-tailed Wilcoxon test was used with a significance level of 0.01.

The transcript fragments were assembled using StringTie software without the use of the GTF annotation file, independently for the GSP1 and GSP2 primers. The default -i flag (0.01) was employed during the assembly of the StringTie fragments. For each primer pair, the total assembly of all submitted *SLC4A11* transcript fragments was conducted using the StringTie-merge function. For assembled 5’ end quantification, the Bowtie2 program (2.3.4.1) was employed to perform read alignment on the sequences of the assembled fragments. The SAM files were subsequently sorted and indexed via SAMtools software (version 1.19.2), and deduplication was conducted using UMI-tools to eliminate duplicate reads resulting from PCR amplification. The unpaired reads were processed via SAMtools software to correct the mating and filtering information. The BAM file was filtered using the SAMtools view command to determine the number of fragments belonging to different types for the two primer pairs. A -q 10 filter was applied to select the most uniquely mapped reads. Each read mapped in forward orientation in the BAM file was checked against the given fragment types using the regular expression grep, and the number of matches for each fragment type was recorded. Forward reads that were mapped to fragment 2 and started before the 3’ end of the sequence common to both fragment 2 (assembled by StringTie) and the first exon of transcript v6 were counted as corresponding to transcript v3 with a long 5’ UTR. This was performed using the SAMtools view command with the specified region. Otherwise, reads corresponding to fragment 2 were counted as belonging to canonical transcript v3. The comparison of read fractions corresponding to the same transcript variant between sample groups obtained with the GSP1 and GSP2 primer sets was conducted using a paired two-sided Wilcoxon test. The UCSC genome browser (http://genome.ucsc.edu)^35^, ggplot2 (3.5.0)^66^ and Inkscape were used for visualization of the results.

### Plasmid constructions

For constructions pV6, pV3 and pM36 obtaining cDNA of SLC4A11 isoforms SLC4A11-v2-M36^17^, SLC4A11-v3^17^ and main part of transcript v6 (full-length 5’UTR, followed by CDS SLC4A11-v2-M36 and 3’UTR fragment) were cloned into commercial vector pIRESpuro3 (Takara) by restriction sites NheI and AgeI. cDNA from sample C3 generated during 5’RACE was used. Forward primers used: v2M36_P1L AGTTCGCTAGCTCTCCCACCATGTCGCAGAA for SLC4A11-v2-M36 CDS amplification; v3P1L AGTTCGCTAGCGTGAGTGCGCGAGTGTGCCAT for SLC4A11-v3 CDS; 6and3_UTR1L; AGTTCGCTAGCGGGGTCTGCCTCAGTCGCACA for amplification of transcript v6; common reverse primer: SLC4AgeIrv: GCTTGAGACCGGTCACACCTACACCTCCCCTCA. CopGFP CDS with flanked sequences, contained NheI on 5’end of CDS and AgeI on 3’end of CDS was kindly provided by Didych DA (IBCH RAS, working of Didych DA with CopGFP constructs confirmed by publication Kashkin et al. 2022^71^ ) and then cloned into pIRESpuro3 for transfection efficiency estimation and using as negative control on SLC4A11 expression in Western-blot analysis. Fragments of *SLC4A11* transcripts were amplified using cDNA from donor C3 (Supplementary File 2). The primer 6and3_UTR1L corresponds to the start of transcripts 6 and 3Long. Plasmids containing the target inserts were identified by PCR screening of E. coli TOP10 clones (ThermoFisher) harboring the plasmids, followed by Sanger sequencing of the insert region. Primers used for screening (seqCMV_F1 and seqSLC_R1) and Sanger sequencing (all primers prefixed “seq” and C2_R1) are listed in Supplementary Table S1. All constructs contained the synonymous benign (ClinVar) rs3803956 (C > T), with a population allele frequency of ~ 0.16633^72,73^ in the SLC4A11 CDS.

### Culture and transfection of HEK293 cells

The cultivation of the HEK293 cells was undertaken in Dulbecco’s modified Eagle medium (DMEM F12), which was enriched with 10% (v/v) foetal bovine serum and an additional 2 mM L-glutamine. The cells were maintained at a temperature of 37 °C in a 5% CO₂ air environment.

The transfection process was executed by employing the Lipofectamine 3000 reagent (Invitrogen; Waltham, Massachusetts, USA), in accordance with manufacturer’s guidelines. The efficiency of transfection was determined by calculating the percentage of green fluorescent protein (GFP)-positive cells relative to the total cell population, using an inverted fluorescence microscope. Transfection was performed in two replicates, and its efficiency was assessed to be 60–70% in both.

### Immunoblots

The cells were detached from a 6-well plate using 0.25% w/v Trypsin-EDTA (Thermo Fisher Scientific; Waltham, Massachusetts, USA). Lysates were prepared using 2x SDS-PAGE Loading Buffer (Non-Reduced) (G2032-1ML, Servicebio) supplemented with beta-mercaptoethanol to a concentration of 5%. Samples were incubated at 65 °C for 5 min, then rapidly chilled on ice for at least 2 min. Cell lysates were homogenized using QIAshredder (Qiagen; Hilden, Germany). Samples were equalized by cell count per µl (the sample volume was calculated based on the expectation that 1 µL of the sample contains 5 × 10^5 cells).

Protein electrophoresis was performed under denaturing conditions using a 5% stacking gel and 7.5% separating gel with a Tris-glycine buffer at 100 V and 10 mA for 1 h. Precision Plus Protein Dual Color 10–250 (Bio-Rad; designated L1 on Fig. 8) and Rav-10 (Biolabmix; designated L2) ladders were loaded with samples. The subsequent implementation of a wet transfer to a PVDF membrane was conducted within a Mini-PROTEAN Tetra chamber (Bio-Rad; Hercules, California, USA). The transfer conditions were set at 350 mA, and a duration of 50 min. Non-specific binding sites were blocked using EveryBlot Blotting Buffer (Bio-Rad; Hercules, California, USA) for a period of 30 min. In the context of the Western blot analysis, the primary antibodies employed included b-actin (Abcam, Cambridge, UK; Cat#ab8226), with a final concentration of 0.5 µg/ml, and SLC4A11 (Sigma-Aldrich, Burlington, Massachusetts, USA; Cat# ABN1718), with a final concentration of 1.25 µg/ml. Secondary antibodies were utilised at a dilution of 1:25000, employing goat anti-rabbit IgG conjugated with horseradish peroxidase (Goat Anti-Rabbit IgG H&L (HRP) preadsorbed; Abcam, Cambridge, UK; Cat# ab97080) and goat anti-mouse IgG conjugated with horseradish peroxidase (Goat Anti-Mouse IgG H&L (HRP) preadsorbed; Abcam, Cambridge, UK; Cat# ab97040). Immunoblots were visualized using Clarity ECL Western Blotting Substrates (Bio-Rad; Hercules, California, USA). The imaging of the membranes was performed using the ChemiDoc™ MP Imaging System (Bioradiations).

### Detection of full-length transcript v6 in different samples of corneal endothelial RNA

PCR was performed on 1ul of cDNA from samples C3, C6 and C63 generated during 5’RACE; primers sequences provided in Supplementary Table S1. Forward primers specific to transcript variants were: v6 (and X7) - SLCv6L, v3 (and all transcripts from group 3 as v3Long) - SLC_allv3L, v2/NR_174471 - SLCv2/NR174L, v2/NR_174471 - SLCv1L/NR174L, common reverse primer - SLC_gsp3. After 32 amplification cycles, 2 µL of each reaction was loaded onto a 1.2% agarose gel.

## Conclusions

The protein-coding transcript 6 of the *SLC4A11* gene, as well as transcript 3, which has an elongated 5’ UTR, have been shown to be highly expressed in the corneal endothelium. Both transcripts share a common transcription initiation start. These findings provide new insights into the regulation of *SLC4A11* expression and the functional consequences of certain genomic variants identified in patients with FECD. Our findings also help resolve some controversies in the literature.

## Supplementary Information

Below is the link to the electronic supplementary material.

Supplementary Material 1

Supplementary Material 2

Supplementary Material 3

Supplementary Material 4

Supplementary Material 5

Supplementary Material 6

Supplementary Material 7

Supplementary Material 8

## Data Availability

Corneal endothelium transcriptomes from FECD patients and donors were uploaded to the NCBI Sequence Read Archive under Accession Nos. PRJNA524323 and PRJNA1146429. (https://www.ncbi.nlm.nih.gov/bioproject/PRJNA524323 and https://www.ncbi.nlm.nih.gov/bioproject/PRJNA1146429). Keratoconic and donor corneal sample transcriptomes are available at the NCBI Sequence Read Archive under Accession No. PRJNA1184491 (https://www.ncbi.nlm.nih.gov/bioproject/PRJNA1184491). The results of the sequencing of 5’ RACE products via the Illumina MiSeq platform are available in the NCBI Sequence Read Archive under Accession No. PRJNA1189328 (https://www.ncbi.nlm.nih.gov/bioproject/PRJNA1189328).

## References

[1] Gröger, N. et al. SLC4A11 prevents osmotic imbalance leading to corneal endothelial dystrophy, deafness, and polyuria. *J. Biol. Chem.* **285**, 14467–14474 (2010). 20185830 10.1074/jbc.M109.094680 PMC2863209

[2] Wieben, E. D. et al. A common trinucleotide repeat expansion within the transcription factor 4 (TCF4, E2-2) gene predicts fuchs corneal dystrophy. *PLoS One*. **7**, e49083 (2012). 23185296 10.1371/journal.pone.0049083 PMC3504061

[3] Mootha, V. V., Gong, X., Ku, H. C. & Xing, C. Association and familial segregation of CTG18. 1 trinucleotide repeat expansion of TCF4 gene in fuchs’ endothelial corneal dystrophy. *Investig. Ophthalmol. Vis. Sci.* **55**, 33–42 (2014). 24255041 10.1167/iovs.13-12611 PMC3880006

[4] Vithana, E. N. et al. SLC4A11 mutations in fuchs endothelial corneal dystrophy. *Hum. Mol. Genet.* **17**, 656–666 (2008). 18024964 10.1093/hmg/ddm337

[5] Riazuddin, S. A. et al. Missense mutations in the sodium borate cotransporter SLC4A11 cause late-onset fuchs corneal dystrophy a. *Hum. Mutat.* **31**, 1261–1268 (2010). 20848555 10.1002/humu.21356 PMC2970683

[6] Tsedilina, T. R., Sharova, E., Iakovets, V. & Skorodumova, L. O. Systematic review of SLC4A11, ZEB1, LOXHD1, and AGBL1 variants in the development of fuchs’ endothelial corneal dystrophy. *Front. Med.* **10**, 1153122 (2023). 10.3389/fmed.2023.1153122 PMC10333596 37441688

[7] Fautsch, M. P. et al. TCF4-mediated fuchs endothelial corneal dystrophy: Insights into a common trinucleotide repeat-associated disease. *Prog. Retin. Eye Res.* **81**, 100883 (2021). 32735996 10.1016/j.preteyeres.2020.100883 PMC7988464

[8] Liu, S. et al. Genetic and demographic determinants of fuchs endothelial corneal dystrophy risk and severity. *JAMA Ophthalmol.* **143**, 338–347 (2025). 40079965 10.1001/jamaophthalmol.2025.0109 PMC11907363

[9] Vithana, E. N. et al. Mutations in sodium-borate cotransporter SLC4A11 cause recessive congenital hereditary endothelial dystrophy (CHED2). *Nat. Genet.* **38**, 755–757 (2006). 16767101 10.1038/ng1824

[10] Jiao, X. et al. Autosomal recessive corneal endothelial dystrophy (CHED2) is associated with mutations in SLC4A11. *J. Med. Genet.* **44**, 64–68 (2007). 16825429 10.1136/jmg.2006.044644 PMC2597914

[11] Sultana, A., Garg, P., Ramamurthy, B., Vemuganti, G. K. & Kannabiran, C. Mutational spectrum of the SLC4A11 gene in autosomal recessive congenital hereditary endothelial dystrophy. *Mol. Vis.* **13**, 1327–1332 (2007). 17679935

[12] Wieben, E. D. et al. Gene expression and missplicing in the corneal endothelium of patients with a TCF4 trinucleotide repeat expansion without fuchs’ endothelial corneal dystrophy. *Investig. Ophthalmol. Vis. Sci.* **60**, 3636–3643 (2019). 31469403 10.1167/iovs.19-27689 PMC6716950

[13] De Roo, A. K., Wouters, J., Govaere, O., Foets, B. & Oord van den, J. J. Identification of circulating fibrocytes and dendritic derivatives in corneal endothelium of patients with fuchs’ dystrophy. *Investig. Ophthalmol. Vis. Sci.* **58**, 670–681 (2017). 28135362 10.1167/iovs.16-20880

[14] Parker, M. D., Ourmozdi, E. P. & Tanner, M. J. Human BTR1, a new bicarbonate transporter superfamily member and human AE4 from kidney. *Biochem. Biophys. Res. Commun.* **282**, 1103–1109 (2001). 11302728 10.1006/bbrc.2001.4692

[15] Kao, L. et al. SLC4A11-c functions as a DIDS-stimulatable H+ (OH-) permeation pathway: Partial correction of R109H mutant transport. *Am. J. Physiology-Cell Physiol.* **308**, C176–C188 (2015). 10.1152/ajpcell.00271.2014 PMC4297769 25394471

[16] Hara, S., Tsujikawa, M., Kawasaki, S. & Nishida, K. Homeostasis of SLC4A11 protein is mediated by endoplasmic reticulum-associated degradation. *Exp. Eye Res.* **188**, 107782 (2019). 31491427 10.1016/j.exer.2019.107782

[17] Malhotra, D., Loganathan, S. K., Chiu, A. M., Lukowski, C. M. & Casey, J. R. Human corneal expression of SLC4A11, a gene mutated in endothelial corneal dystrophies. *Sci. Rep.* **9**, 9681 (2019). 31273259 10.1038/s41598-019-46094-y PMC6609610

[18] Kao, L. et al. Multifunctional ion transport properties of human SLC4A11: Comparison of the SLC4A11-b and SLC4A11-c variants. *Am. J. Physiology-Cell Physiol.* **311**, C820–C830 (2016). 10.1152/ajpcell.00233.2016 PMC5130583 27581649

[19] Quade, B. N., Marshall, A. & Parker, M. D. Corneal dystrophy mutations R125H and R804H disable SLC4A11 by altering the extracellular pH dependence of the intracellular pK that governs h+ (OH-) transport. *Am. J. Physiology-Cell Physiol.* **323**, C990–C1002 (2022). 10.1152/ajpcell.00221.2022 PMC9484998 35993514

[20] Chung, D. D. et al. Investigation of the functional impact of CHED-and FECD4-associated SLC4A11 mutations in human corneal endothelial cells. *Plos one*. **19**, e0296928 (2024). 38252645 10.1371/journal.pone.0296928 PMC10802951

[21] Choi, M. & Bonanno, J. A. Mitochondrial targeting of the ammonia-sensitive uncoupler SLC4A11 by the chaperone-mediated carrier pathway in corneal endothelium. *Investig. Ophthalmol. Vis. Sci.* **62**, 4–4 (2021). 10.1167/iovs.62.12.4 PMC8434753 34499705

[22] Hemadevi, B. et al. Identification of mutations in the SLC4A11 gene in patients with recessive congenital hereditary endothelial dystrophy. *Arch. Ophthalmol.* **126**, 700–708 (2008). 18474783 10.1001/archopht.126.5.700

[23] Guha, S. & Roy, S. Enhanced expression of SLC4A11 by tert-butylhydroquinone is mediated by direct binding of Nrf2 to the promoter of SLC4A11. *Free Radic. Biol. Med.* **167**, 299–306 (2021). 33744340 10.1016/j.freeradbiomed.2021.03.006

[24] Nikitina, A. S. et al. Dataset on transcriptome profiling of corneal endothelium from patients with fuchs endothelial corneal dystrophy. *Data brief.* **25**, 104047 (2019). 31205988 10.1016/j.dib.2019.104047 PMC6558234

[25] Chen, Y. et al. Identification of novel molecular markers through transcriptomic analysis in human fetal and adult corneal endothelial cells. *Hum. Mol. Genet.* **22**, 1271–1279 (2013). 23257286 10.1093/hmg/dds527 PMC3596846

[26] Chu, Y. et al. Analyzing pre-symptomatic tissue to gain insights into the molecular and mechanistic origins of late-onset degenerative trinucleotide repeat disease. *Nucleic Acids Res.* **48**, 6740–6758 (2020). 32463444 10.1093/nar/gkaa422 PMC7337964

[27] He, H. et al. Transcriptome analysis discloses dysregulated genes in normal appearing tumor-adjacent thyroid tissues from patients with papillary thyroid carcinoma. *Sci. Rep.* **11**, 14126 (2021). 34238982 10.1038/s41598-021-93526-9 PMC8266864

[28] Zhu, X. et al. CXCR2 may serve as a useful index of disease activity in interstitial lung disease associated with primary sjögren’s syndrome. *Front. Mol. Biosci.* **8**, 640779 (2021). 34055876 10.3389/fmolb.2021.640779 PMC8155469

[29] The GTEx portal. (2024). https://gtexportal.org/home/. Accessed 30 apr 2024.

[30] Gu, W. et al. SATB2 preserves colon stem cell identity and mediates ileum-colon conversion via enhancer remodeling. *Cell. Stem Cell.* **29**, 101–115 (2022). 34582804 10.1016/j.stem.2021.09.004 PMC8741647

[31] Devall, M. A. et al. Transcriptomic response to calcium in normal colon organoids is impacted by colon location and sex. *Cancer Prev. Res.* **15**, 679–688 (2022). 10.1158/1940-6207.CAPR-22-0068 PMC9530641 36095330

[32] Quentmeier, H. et al. The LL-100 panel: 100 cell lines for blood cancer studies. *Sci. Rep.* **9**, 8218 (2019). 31160637 10.1038/s41598-019-44491-x PMC6547646

[33] Nakagawa, T. et al. RNA-Seq–based transcriptome analysis of corneal endothelial cells derived from patients with Fuchs endothelial corneal dystrophy. *Sci. Rep.* **13**, 8647 (2023). 37244951 10.1038/s41598-023-35468-y PMC10224979

[34] Still, E. R. & Yuneva, M. O. Hopefully devoted to q: Targeting glutamine addiction in cancer. *Br. J. Cancer*. **116**, 1375–1381 (2017). 28441384 10.1038/bjc.2017.113 PMC5520092

[35] Perez, G. et al. The UCSC genome browser database: 2025 update. *Nucleic Acids Research* gkae974 (2024). 10.1093/nar/gkae974 PMC11701590 39460617

[36] Calvo, S. E., Pagliarini, D. J. & Mootha, V. K. Upstream open reading frames cause widespread reduction of protein expression and are polymorphic among humans. *Proceedings of the National Academy of Sciences* 106, 7507–7512 (2009). 10.1073/pnas.0810916106 PMC2669787 19372376

[37] Ye, Y. et al. Analysis of human upstream open reading frames and impact on gene expression. *Hum. Genet.* **134**, 605–612 (2015). 25800702 10.1007/s00439-015-1544-7

[38] Barbosa, C., Peixeiro, I. & Romão, L. Gene expression regulation by upstream open reading frames and human disease. *PLoS Genet.* **9**, e1003529 (2013). 23950723 10.1371/journal.pgen.1003529 PMC3738444

[39] Kozak, M. An analysis of 5’-noncoding sequences from 699 vertebrate messenger RNAs. *Nucleic Acids Res.* **15**, 8125–8148 (1987). 3313277 10.1093/nar/15.20.8125 PMC306349

[40] Kozak, M. Evaluation of the scanning model for initiation of protein synthesis in eucaryotes. *Cell* **22**, 7–8 (1980). 7000367 10.1016/0092-8674(80)90148-8

[41] Andreev, D. E. et al. Non-AUG translation initiation in mammals. *Genome Biol.* **23**, 111 (2022). 35534899 10.1186/s13059-022-02674-2 PMC9082881

[42] Loganathan, S. K., Schneider, H. P., Morgan, P. E., Deitmer, J. W. & Casey, J. R. Functional assessment of SLC4A11, an integral membrane protein mutated in corneal dystrophies. *Am. J. Physiology-Cell Physiol.* **311**, C735–C748 (2016). 10.1152/ajpcell.00078.2016 PMC5130586 27558157

[43] Maston, G. A., Evans, S. K. & Green, M. R. Transcriptional regulatory elements in the human genome. *Annu. Rev. Genomics Hum. Genet.* **7**, 29–59 (2006). 16719718 10.1146/annurev.genom.7.080505.115623

[44] Deaton, A. M. & Bird, A. CpG islands and the regulation of transcription. *Genes Dev.* **25**, 1010–1022 (2011). 21576262 10.1101/gad.2037511 PMC3093116

[45] Haberle, V. & Stark, A. Eukaryotic core promoters and the functional basis of transcription initiation. *Nat. Rev. Mol. Cell Biol.* **19**, 621–637 (2018). 29946135 10.1038/s41580-018-0028-8 PMC6205604

[46] Frausto, R. F., Le, D. J. & Aldave, A. J. Transcriptomic analysis of cultured corneal endothelial cells as a validation for their use in cell replacement therapy. *Cell Transplant.* **25**, 1159–1176 (2016). 26337789 10.3727/096368915X688948 PMC4775465

[47] Malhotra, D. et al. Defective cell adhesion function of solute transporter, SLC4A11, in endothelial corneal dystrophies. *Hum. Mol. Genet.* **29**, 97–116 (2020). 31691803 10.1093/hmg/ddz259

[48] Chung, D. D. et al. Investigation of the functional impact of CHED-and FECD4-associated SLC4A11 mutations in human corneal endothelial cells. *Plos one*. **19**, e0296928 (2024). 38252645 10.1371/journal.pone.0296928 PMC10802951

[49] Cabahug, V. L. O. et al. Mutational analysis of the SLC4A11 gene in a (filipino) family with congenital hereditary endothelial dystrophy. *J. Clin. Translational Ophthalmol.* **2**, 34–46 (2024).

[50] Deplancke, B., Alpern, D. & Gardeux, V. The genetics of transcription factor DNA binding variation. *Cell* **166**, 538–554 (2016). 27471964 10.1016/j.cell.2016.07.012

[51] Skorodumova, L. O. et al. CTG18. 1 expansion is the best classifier of late-onset fuchs’ corneal dystrophy among 10 biomarkers in a cohort from the european part of russia. *Investig. Ophthalmol. Vis. Sci.* **59**, 4748–4754 (2018). 30267097 10.1167/iovs.18-24590

[52] Skorodumova, L. O. et al. Rare single nucleotide variants in COL5A1 promoter do not play a major role in keratoconus susceptibility associated with rs1536482. *BMC Ophthalmol.* **21**, 1–9 (2021). 34625056 10.1186/s12886-021-02128-6 PMC8501560

[53] Matz, M. et al. Amplification of cDNA ends based on template-switching effect and step-out PCR. *Nucleic Acids Res.* **27**, 1558–1560 (1999). 10037822 10.1093/nar/27.6.1558 PMC148354

[54] Mamedov, I. Z. et al. Preparing unbiased t-cell receptor and antibody cDNA libraries for the deep next generation sequencing profiling. *Front. Immunol.* **4**, 456 (2013). 24391640 10.3389/fimmu.2013.00456 PMC3870325

[55] Kivioja, T. et al. Counting absolute numbers of molecules using unique molecular identifiers. *Nat. Methods*. **9**, 72–74 (2012). 10.1038/nmeth.1778 22101854

[56] Picelli, S. et al. Full-length RNA-seq from single cells using smart-seq2. *Nat. Protoc.* **9**, 171–181 (2014). 24385147 10.1038/nprot.2014.006

[57] Gobbini, A., Bandera, A., Grifantini, R., Abrignani, S. & Notarbartolo, S. Protocol for the detection of defined t cell clones in a heterogeneous cell population. *STAR. protocols*. **5**, 102787 (2024). 38141168 10.1016/j.xpro.2023.102787 PMC10783555

[58] Zhang, W., Ogando, D. G., Bonanno, J. A. & Obukhov, A. G. Human SLC4A11 is a novel NH3/h + co-transporter. *J. Biol. Chem.* **290**, 16894–16905 (2015). 26018076 10.1074/jbc.M114.627455 PMC4505435

[59] The GTEx portal. 2024. https://gtexportal.org/home/. Accessed 30 apr 2024.

[60] Pertea, G. & Pertea, M. GFF utilities: GffRead and GffCompare. F1000Research 9, (2020). 10.12688/f1000research.23297.1 PMC7222033 32489650

[61] Pertea, M. et al. StringTie enables improved reconstruction of a transcriptome from RNA-seq reads. *Nat. Biotechnol.* **33**, 290–295 (2015). 25690850 10.1038/nbt.3122 PMC4643835

[62] Patro, R., Duggal, G., Love, M. I., Irizarry, R. A. & Kingsford, C. Salmon provides fast and bias-aware quantification of transcript expression. *Nat. Methods*. **14**, 417–419 (2017). 28263959 10.1038/nmeth.4197 PMC5600148

[63] Team, R. *RStudio team (2020). RStudio: Integrated development for r. RStudio* (PBC, boston, 2020).

[64] Soneson, C., Love, M. I. & Robinson, M. D. Differential analyses for RNA-seq: Transcript-level estimates improve gene-level inferences. *F1000Research* 4, (2015). 10.12688/f1000research.7563.1 PMC4712774 26925227

[65] Robinson, M. D., McCarthy, D. J. & Smyth, G. K. edgeR: A bioconductor package for differential expression analysis of digital gene expression data. *bioinformatics* **26**, 139–140 (2010). 19910308 10.1093/bioinformatics/btp616 PMC2796818

[66] Wickham, H. 2106. ggplot2: Elegant graphics for data analysis.

[67] Consortium, G. The GTEx consortium atlas of genetic regulatory effects across human tissues. *Science* **369**, 1318–1330 (2020). 32913098 10.1126/science.aaz1776 PMC7737656

[68] Lonsdale, J. et al. The genotype-tissue expression (GTEx) project. *Nat. Genet.* **45**, 580–585 (2013). 23715323 10.1038/ng.2653 PMC4010069

[69] Love, M. I., Huber, W. & Anders, S. Moderated estimation of fold change and dispersion for RNA-seq data with DESeq2. *Genome Biol.* **15**, 550 (2014). 25516281 10.1186/s13059-014-0550-8 PMC4302049

[70] Gleason, A. C., Ghadge, G., Chen, J., Sonobe, Y. & Roos, R. P. Machine learning predicts translation initiation sites in neurologic diseases with nucleotide repeat expansions. *PLoS One*. **17**, e0256411 (2022). 35648796 10.1371/journal.pone.0256411 PMC9159584

[71] Kashkin, K. N. et al. Efficient selection of enhancers and promoters from MIA PaCa-2 pancreatic cancer cells by ChIP-lentiMPRA. *Int. J. Mol. Sci.* **23**, 15011 (2022). 36499347 10.3390/ijms232315011 PMC9740945

[72] Landrum, M. J. et al. Public archive of relationships among sequence variation and human phenotype. *Nucleic Acids Res.* **42**, D980–D985 (2014). ClinVar. 24234437 10.1093/nar/gkt1113 PMC3965032

[73] National center for biotechnology information. ClinVar; [VCV000262002.21], https://www.ncbi.nlm.nih.gov/clinvar/variation/VCV000262002.21 (2025). (accessed dec. 14.

