# Supplementary material for: High expression of the underexplored SLC4A11 protein-coding transcript is specific to the corneal endothelium
Source: Sci Rep. 2026 May 11;16:21446. doi: 10.1038/s41598-026-51987-w (PMC13351061; doi:10.1038/s41598-026-51987-w)
Supplement: Supplementary file 5 — Supplementary Material 5 [file 41598_2026_51987_MOESM5_ESM.docx]

Supplementary Table S1. Oligonucleotides sequences.

| Oligo-  nucleotide name | Oligonucleotide sequence |
| --- | --- |
| TSO | AAGCAGTGGTATCAACGCAGAGTSWNNNSWNNNSWNNNSWGTCATrGrGrGrGrGlna |
| Long step-out primer | CTAATACGACTCACTATAGGGCAAGCAGTGGTATCAACGCAGAGT |
| Short step-out primer2 | CTAATACGACTCACTATAGGGC |
| GSP1out | GAATCCTCGAAGTATCCATTC |
| GSP1in | CGAAGTATCCATTCTGCGACA |
| GSP2out | GGATACTCTCGCCAGACACGA |
| GSP2in | GGAGGAGTTGGCAGTGTCGAA |
| v2M36_P1L | AGTTCGCTAGCTCTCCCACCATGTCGCAGAA |
| v3P1L | AGTTCGCTAGCGTGAGTGCGCGAGTGTGCCAT |
| 6and3_UTR1L | AGTTCGCTAGCGGGGTCTGCCTCAGTCGCACA |
| SLC4AgeIrv | GCTTGAGACCGGTCACACCTACACCTCCCCTCA |
| seqCMV_F1 | TCCAAGTCTCCACCCCATTG |
| seqSLC_R1 | AGGATGACGAACCGAACCTC |
| seqSLC_F2 | ACAATGAGCCCAACTGCAAC |
| seqSLC_R2 | ATGATCTCCTCCGTCGACCT |
| seqSLC_F3 | CCTTGGACTTCACTGATGGCA |
| seqSLC_R3 | GCCACCAAGTTCTGCTCGAT |
| seqSLC_F4 | CCGGGAAATCGAGATGAGCAA |
| C2_R1 | GAGGGAGTACTCACCCCAACAG |
| SLCv6L | GCAGCCTCCCGCGGAAAACTC |
| SLC_allv3L | GTGAGTGCGCGAGTGTGCCAT |
| SLCv2/NR174L | AAGAGCGAAGGAATGAGCCA |
| SLCv1L/NR174L | CGCCTGCAATGGGCGTTTATG |
| SLC_gsp3 | AGCAGTCACCCACACACCTAC |

N - any base, W - A/T, S - G/C, rG - G in ribonucleoside, rGlna - G in ribonucleoside with LNA (locked nucleotide acid modification)

Supplementary Table S2. Statistical significance (p-value) of differences in the expression levels of each of the five most expressed transcript variants compared to variant 2 within the same group and dataset (Wilcoxon test, paired, one sided).

| Transcript | Endo1, control group | Endo1, FECD group | Endo2, Control group | Endo2, FECD group |
| --- | --- | --- | --- | --- |
| ENST00000642402 | 3.91E-03 | 6.10E-05 | 3.05E-05 | 9.77E-04 |
| ENST00000644862 | 3.91E-03 | 1.05E-03 | 3.05E-05 | 9.77E-04 |
| NM_001400277 | 7.81E-03 | 1.22E-04 | 6.10E-05 | 9.77E-04 |
| ENST00000437836 | 7.81E-03 | 6.10E-05 | 3.05E-05 | 9.77E-04 |
| NR_174471 | 7.81E-03 | 1.83E-04 | 3.05E-05 | 9.77E-04 |

Supplementary Table S3. Statistical significance (p-value) of differences in the expression of the five most represented transcripts or transcript variant 2 between the FECD and control groups in both datasets (Wilcoxon test, non paired, two sided).

| Transcript | Dataset | |
| --- | --- | --- |
|  | endo1 | endo2 |
| v3 | 0.0502 | 0.9783 |
| v6short | 0.0698 | 0.2380 |
| v6 | 0.0292 | 0.2164 |
| E_7836 | 0.6163 | 0.8065 |
| NR_174471 | 0.6642 | 0.4611 |
| v2 | 0.6319 | 0.1992 |

Supplementary Table S4. Statistically significant differences (p-value) in the fractions of TSS detected in all of the analysed initiation regions between the FECD and groups (Wilcoxon test, non paired, two sided).

| Transcript | GSP type | |
| --- | --- | --- |
|  | GSP1 | GSP2 |
| v2 | 0.031746 | 0.095238 |
| NR_174471 | 0.015873 | 0.007937 |
| E_7836 | 0.600402 | 1 |
| v3 | 0.84127 | 0.309524 |
| v6 | 0.055556 | 0.547619 |

Supplementary Table S5. The statistical significance of the differences (p-value) in TSS fractions corresponded to the initiation region of transcript 6 and to other initiation regions in each sample (Chi-square test).

| GSP variant | Sample | variant 2 | NR_174471 | E_7836 | variant 3 |
| --- | --- | --- | --- | --- | --- |
| GSP1 | c203 | 0 | 0 | 0 | 0 |
|  | c206 | 0 | 0 | 0 | 0 |
|  | c208 | 0 | 0 | 0 | 0 |
|  | c263 | 0 | 0 | 0 | 0 |
|  | c264 | 1.27E-254 | 4.32E-229 | 2.64E-248 | 1.06E-182 |
|  | dfu224 | 2.33E-108 | 1.42E-117 | 4.89E-169 | 2.05E-153 |
|  | dfu226 | 0 | 1.07E-283 | 0 | 0 |
|  | dfu270 | 0 | 0 | 0 | 8.39E-303 |
|  | dfu271 | 0 | 0 | 0 | 0 |
|  | dfu280 | 0 | 9.23E-251 | 0 | 4.18E-237 |
| GSP2 | c203 | 0 | 0 | 0 | 0 |
|  | c206 | 0 | 0 | 0 | 0 |
|  | c208 | 0 | 0 | 0 | 0 |
|  | c263 | 0 | 0 | 0 | 0 |
|  | c264 | 5.05E-123 | 3.16E-151 | 7.19E-152 | 5.96E-35 |
|  | dfu224 | 3.86E-148 | 2.04E-131 | 6.59E-180 | 7.42E-100 |
|  | dfu226 | 0 | 0 | 0 | 0 |
|  | dfu270 | 0 | 0 | 0 | 0 |
|  | dfu271 | 0 | 0 | 0 | 0 |
|  | dfu280 | 0 | 0 | 0 | 5.27E-221 |

Supplementary Table S6. Statistical significance (p-value) of differences in TSS fractions among initiation regions of SLC4A11 transcript variants, analyzed using GSP1 primers (Wilcoxon test, paired, two sided)

|  | v2 | NR_174471 | E_7836 | v3 | v6 |
| --- | --- | --- | --- | --- | --- |
| v2 | NaN | 3.71E-02 | 5.80E-02 | 6.45E-02 | 1.95E-03 |
| NR_174471 | 3.71E-02 | NaN | 1.95E-03 | 1.60E-01 | 1.95E-03 |
| E_7836 | 5.80E-02 | 1.95E-03 | NaN | 1.95E-03 | 1.95E-03 |
| v3 | 6.45E-02 | 1.60E-01 | 1.95E-03 | NaN | 1.95E-03 |
| v6 | 1.95E-03 | 1.95E-03 | 1.95E-03 | 1.95E-03 | NaN |

Supplementary Table S7. Statistical significance (p-value) of differences in TSS fractions among initiation regions of SLC4A11 transcript variants, analyzed using GSP2 primers (Wilcoxon test, paired, two sided)

|  | v2 | NR_174471 | E_7836 | v3 | v6 |
| --- | --- | --- | --- | --- | --- |
| v2 | NaN | 2.73E-02 | 1.05E-01 | 1.95E-03 | 1.95E-03 |
| NR_174471 | 2.73E-02 | NaN | 1.95E-02 | 1.95E-02 | 1.95E-03 |
| E_7836 | 1.05E-01 | 1.95E-02 | NaN | 1.95E-03 | 1.95E-03 |
| v3 | 1.95E-03 | 1.95E-02 | 1.95E-03 | NaN | 1.95E-03 |
| v6 | 1.95E-03 | 1.95E-03 | 1.95E-03 | 1.95E-03 | NaN |

Supplementary Table S8. Medians and averages of read fractions corresponded to 5’-end fragments assembled using StringTie.

|  | Median | | Average | | Medians  Ratio, GSP1 and GSP2 | Averages  Ratio, GSP1 and GSP2 | p-val, GSP1 and GSP2 |
| --- | --- | --- | --- | --- | --- | --- | --- |
|  | GSP1 | GSP2 | GSP1 | GSP2 |  |  |  |
| fragm.1, v6 | 0.6422 | 0.6281 | 0.6621 | 0.6146 | 1.0224 | 1.0773 | 4.32e-1 |
| fragm.2, v3 (all 5’UTRs) | 0.3578 | 0.3372 | 0.3379 | 0.3439 | 1.0611 | 0.9826 | 1.0 |
| fragm.3, E_7836 | NA | 0.0063 | NA | 0.0125 | NA | NA | NA |
| fragm.4 | NA | 0.0319 | NA | 0.0290 | NA | NA | NA |
| v3 (Long 5’UTR) | 0.2293 | 0.1800 | 0.2377 | 0.1621 | 1.2739 | 1.4664 | 3.91e-3 |
| v3 (Short 5’UTR) | 0.1044 | 0.1419 | 0.1002 | 0.1818 | 0.7357 | 0.5512 | 1.95e-2 |

Also provided statistically significance of differences between read fractions, obtained with GSP1 and GSP2. Read Fractions were obtained by dividing all reads mapped to fragment on all mapped paired reads from 5’RACE, p-value was obtained by paired two sided Wilcoxon test.
