# Supplementary material for: High expression of the underexplored SLC4A11 protein-coding transcript is specific to the corneal endothelium
Source: Sci Rep. 2026 May 11;16:21446. doi: 10.1038/s41598-026-51987-w (PMC13351061; doi:10.1038/s41598-026-51987-w)
Supplement: Supplementary file 7 — Supplementary Material 7 [file 41598_2026_51987_MOESM7_ESM.pdf]

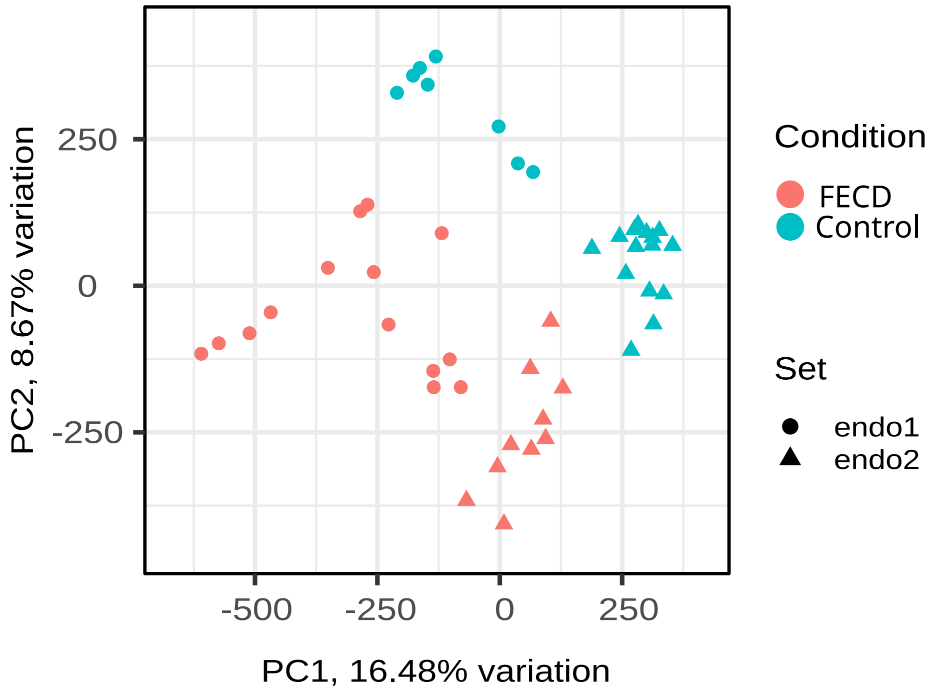

Supplementary Figure S1. Heterogeneity of samples in two transcriptomic datasets (PCA biplot of all transcript expression levels in log2RPKM).

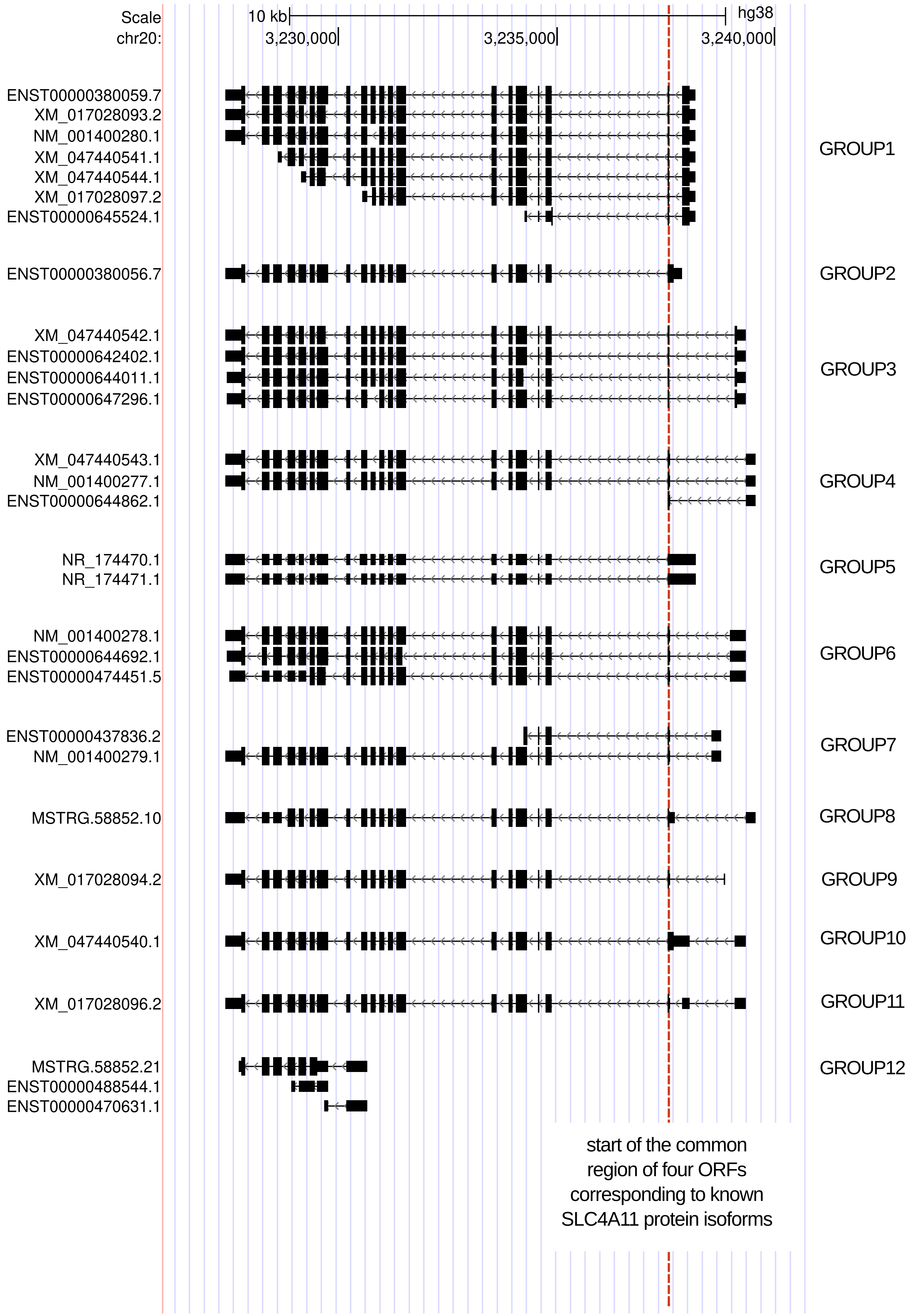

Supplementary Figure S2. Transcript grouping by 5'-ends similarity. The red dotted line marks the start of the common region of four ORFs corresponding to known SLC4A11 protein isoforms.

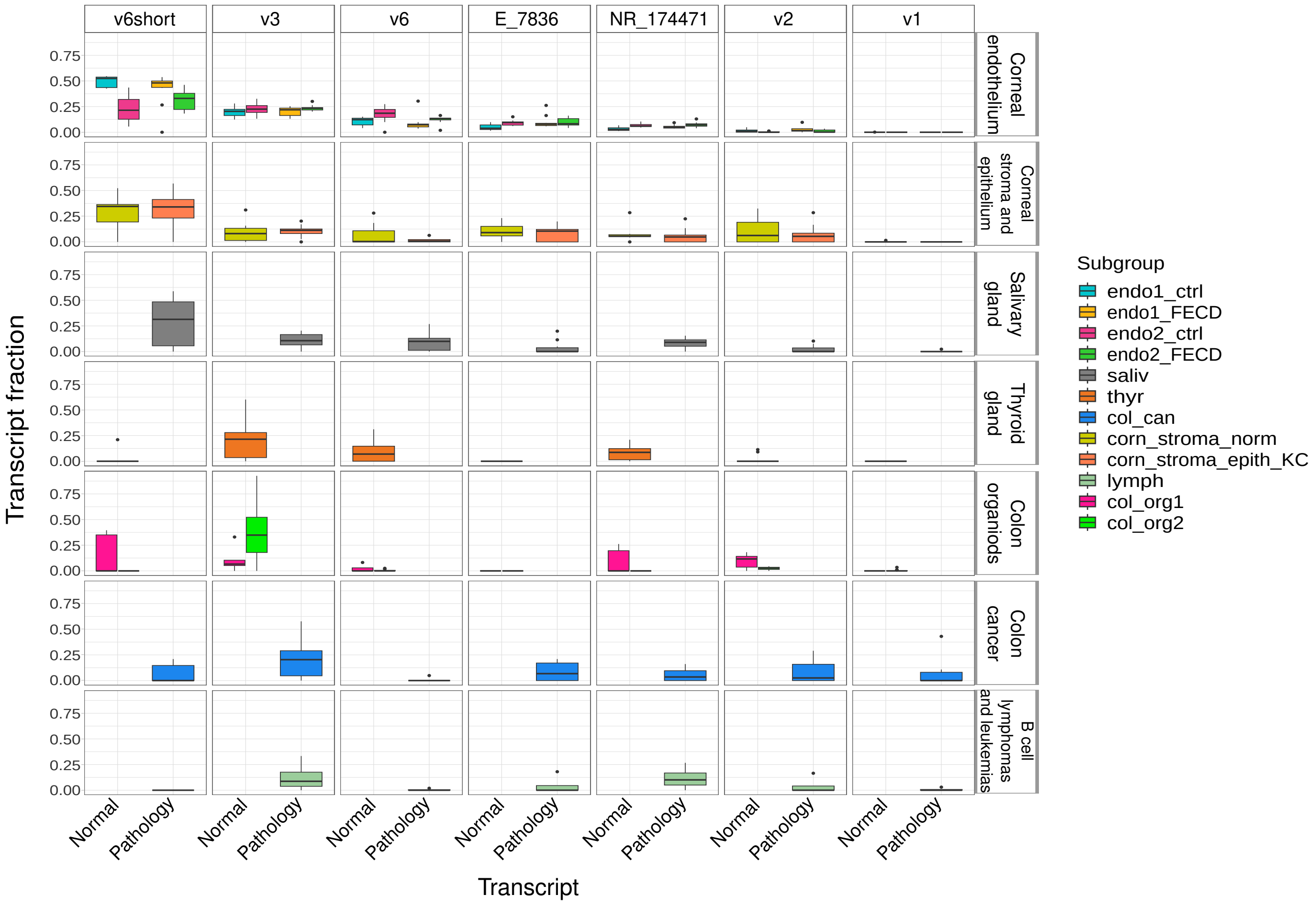

Supplementary Figure S3. Fractions of the SLC4A11 transcripts were the highest in the corneal endothelium in samples combined by tissue, set and the presence or absence of pathology. The following subgroups of samples are shown: endo1_ctrl, control group on endo1 set; endo1_FECD, FECD group of endo1 set; endo2_ctrl, control group of endo2 set; endo2_FECD, FECD group of endo2 set; saliv, samples from saliv set; thyr, samples from thyr set; col_can, samples from col_can set; corn_stroma_norm, normal corneal stroma from kc set; corn_stroma_epith_KC, corneal stroma and corneal stroma with epithelium from patients with keratoconus from kc set; lymph, samples from lymph set; col_org1, samples from col_org1 set; col_org2, samples from col_org2 set.

Although transcripts v3, v6, and v6short exhibited similarly high proportions of total SLC4A11 expression in the corneal endothelial sample groups, some sample groups, such as col_org2 and lymph, expressed v3 but did not express v6 or v6short. Although transcripts v3, v6, and v6short exhibited similarly high proportions of total SLC4A11 expression in the corneal endothelial sample groups, some sample groups, such as col_org2 and lymph, expressed v3 but did not express variants v6 or v6short.

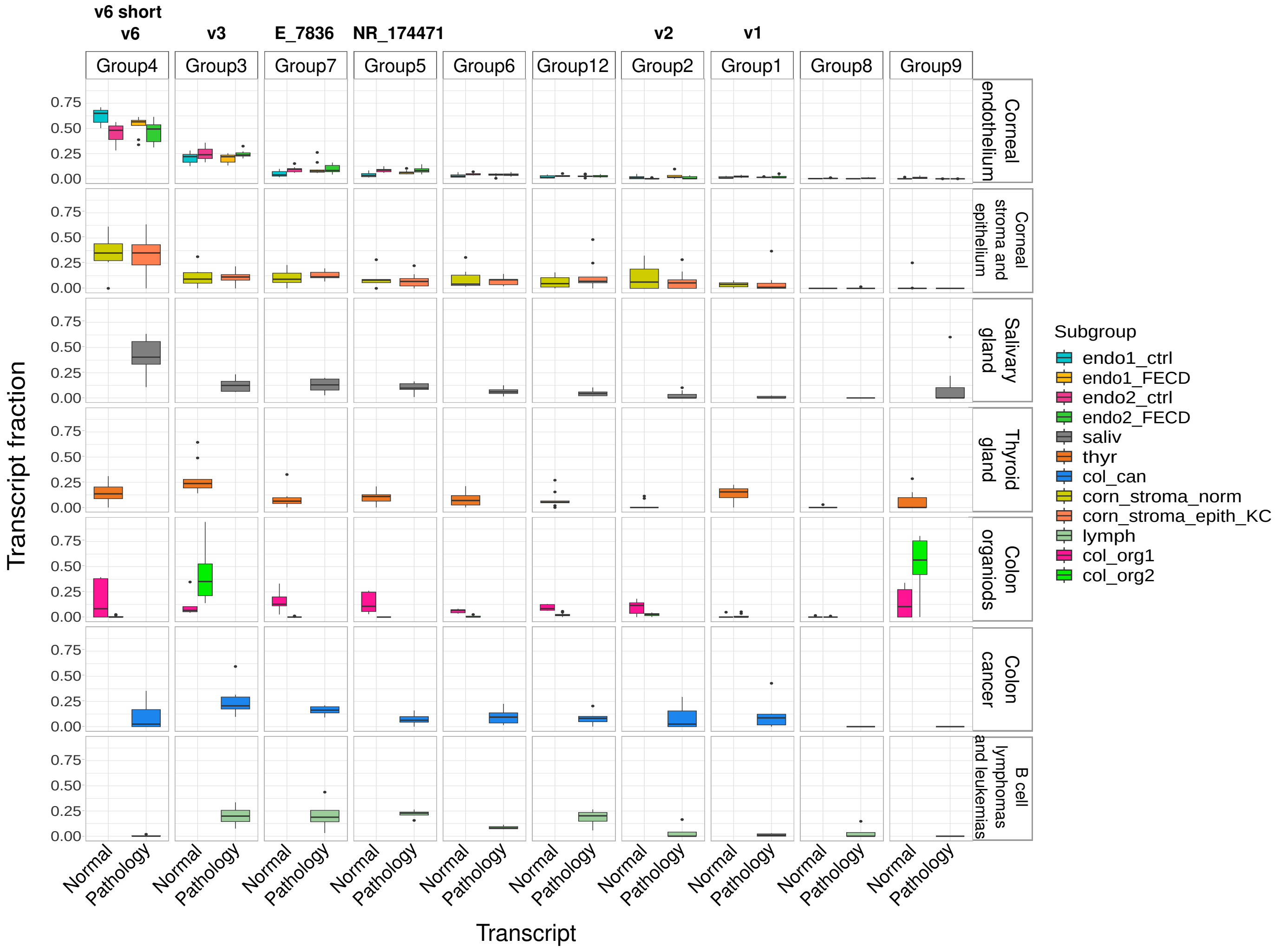

Supplementary Figure S4. Fractions of the SLC4A11 transcript groups from the total gene expression data. Groups of transcripts with maximal fractions of less than 0.1 of total expression among all samples are not represented; v6, v6short, v3, E_7836, NR_174471, v1, and v2 above the group name indicate that this transcript belongs to that group. The following subgroups of samples are shown: endo1_ctrl, control group of the endo1 set; endo1_FECD, FECD group of the endo1 set; endo2_ctrl, control group of the endo2 set; endo2_FECD, FECD group of the endo2 set; saliv, samples from the saliv set; thyr, samples from the thyr set; col_can, samples from the col_can set; corn_stroma_norm, normal corneal stroma from the kc set; corn_stroma_epith_KC, corneal stroma and corneal stroma with epithelium from patients with keratoconus from the kc set; lymph, samples from the lymph set; col_org1, samples from the col_org1 set; and col_org2, samples from the col_org2 set.

Group 4 is the most expressed in corneal endothelium. Group 3 is one of the most represented groups in all analyzed datasets. Among the two sets of transcriptomes (col_org2 and lymph), group 4 had markedly lower expression levels than did group 3.

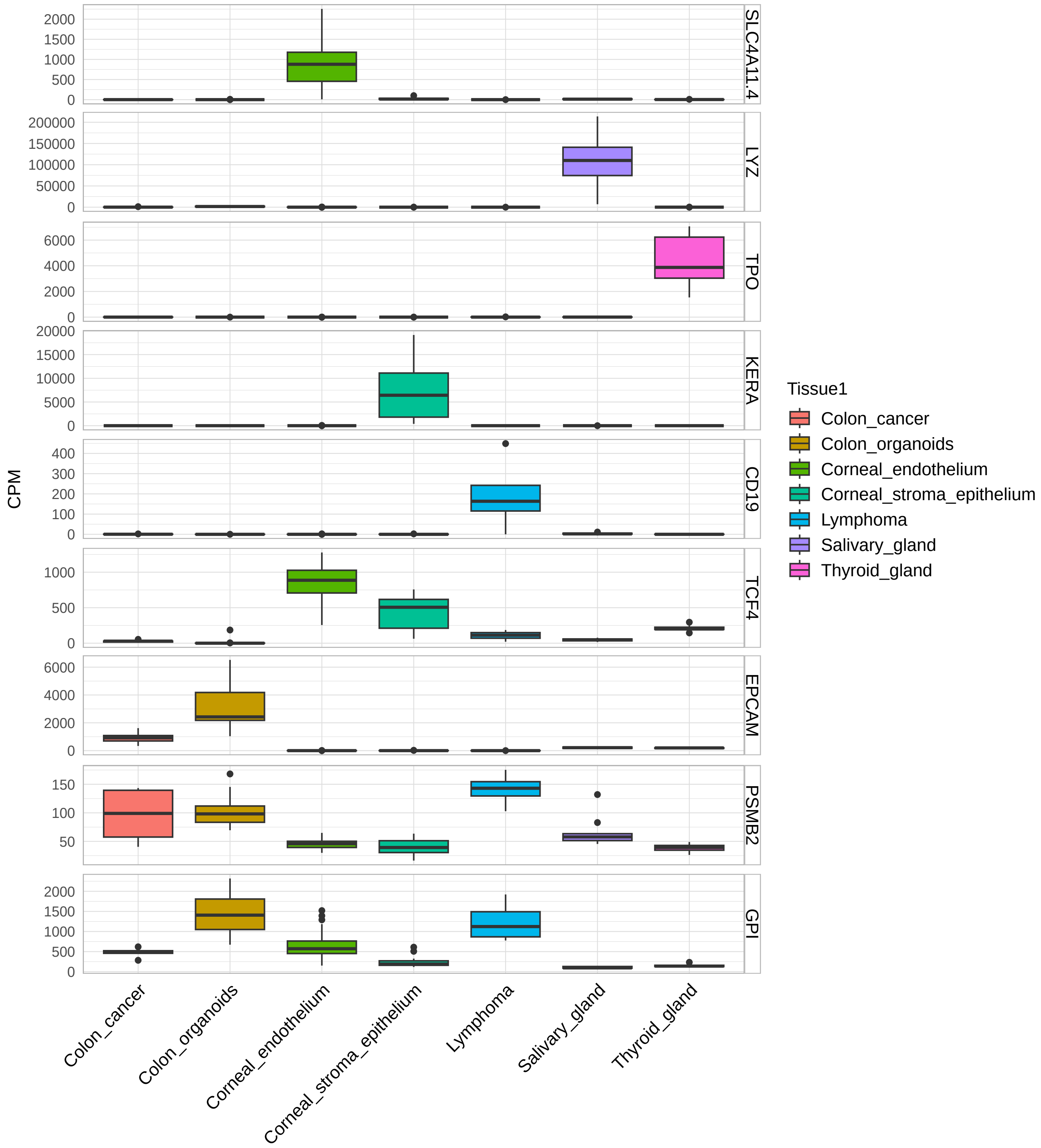

Supplementary Figure S5. Expression of higly tissue specific genes across analyzed datasets. Expression levels are shown in CPM. Specificity of expression: CD19 - B-lymphocytes, including B-cell lymphoma, EPCAM - epithelium, including normal human colorectal epithelium and colorectal cancer cells, KERA - corneal stroma, LYZ - salivary gland, TCF4 - nervous system and cornea, TPO - thyroid gland, GPI and PSMB2 - housekeeping genes (included for comparison).

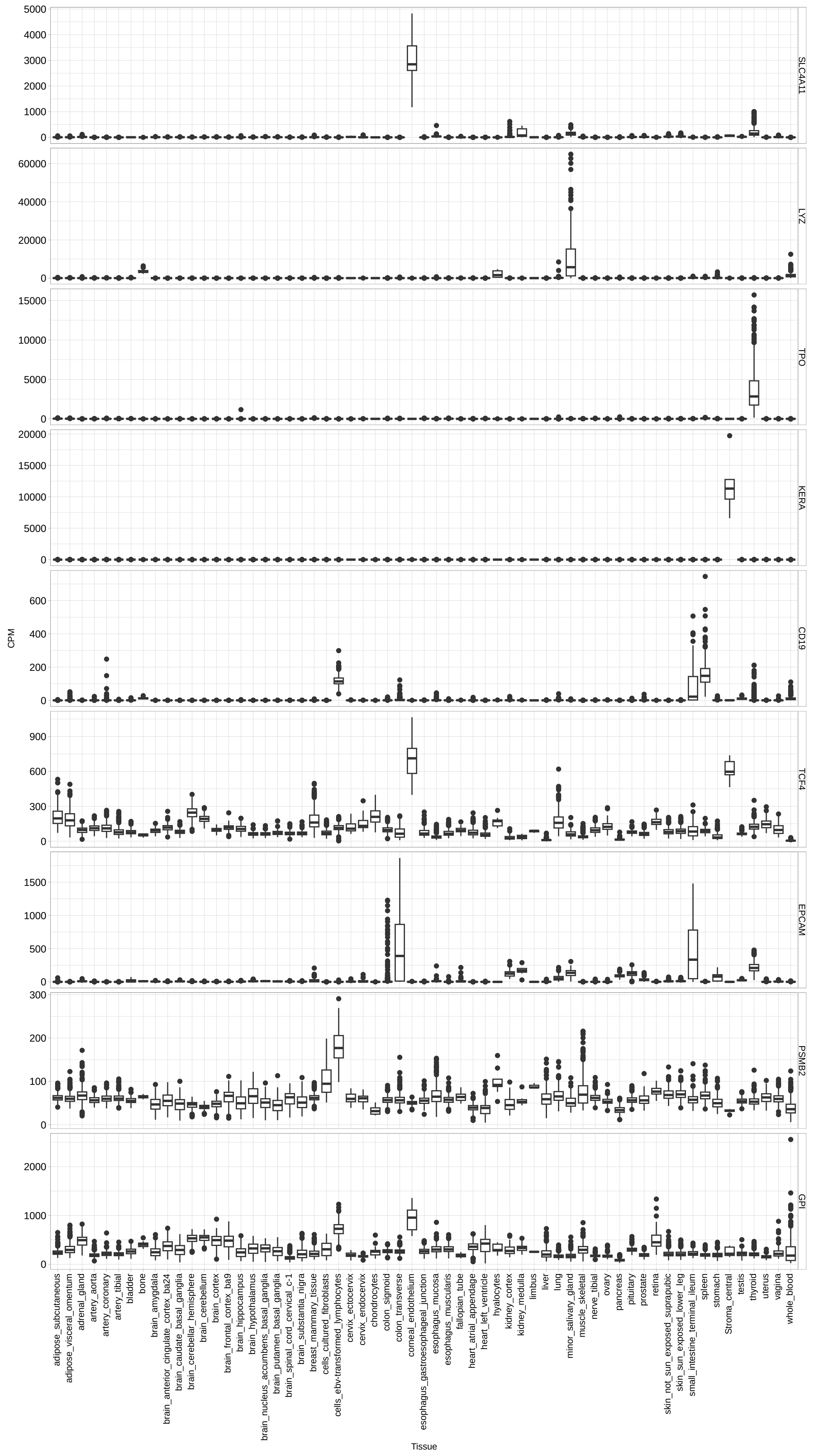

Supplementary Figure S6. Tissue specificity of gene expression: GTEx datasets vs. corneal endothelium. Expression levels are shown in RPKM. Specificity of expression: LYZ - salivary gland, TPO - thyroid gland, KERA - corneal stroma, CD19 - B-lymphocytes, including B-celllymphoma, EPCAM - epithelium, including normal human colorectalepithelium and colorectal cancer cells, TCF4 - nervous system and cornea, GPI and PSMB2 - housekeeping genes (included for comparison).

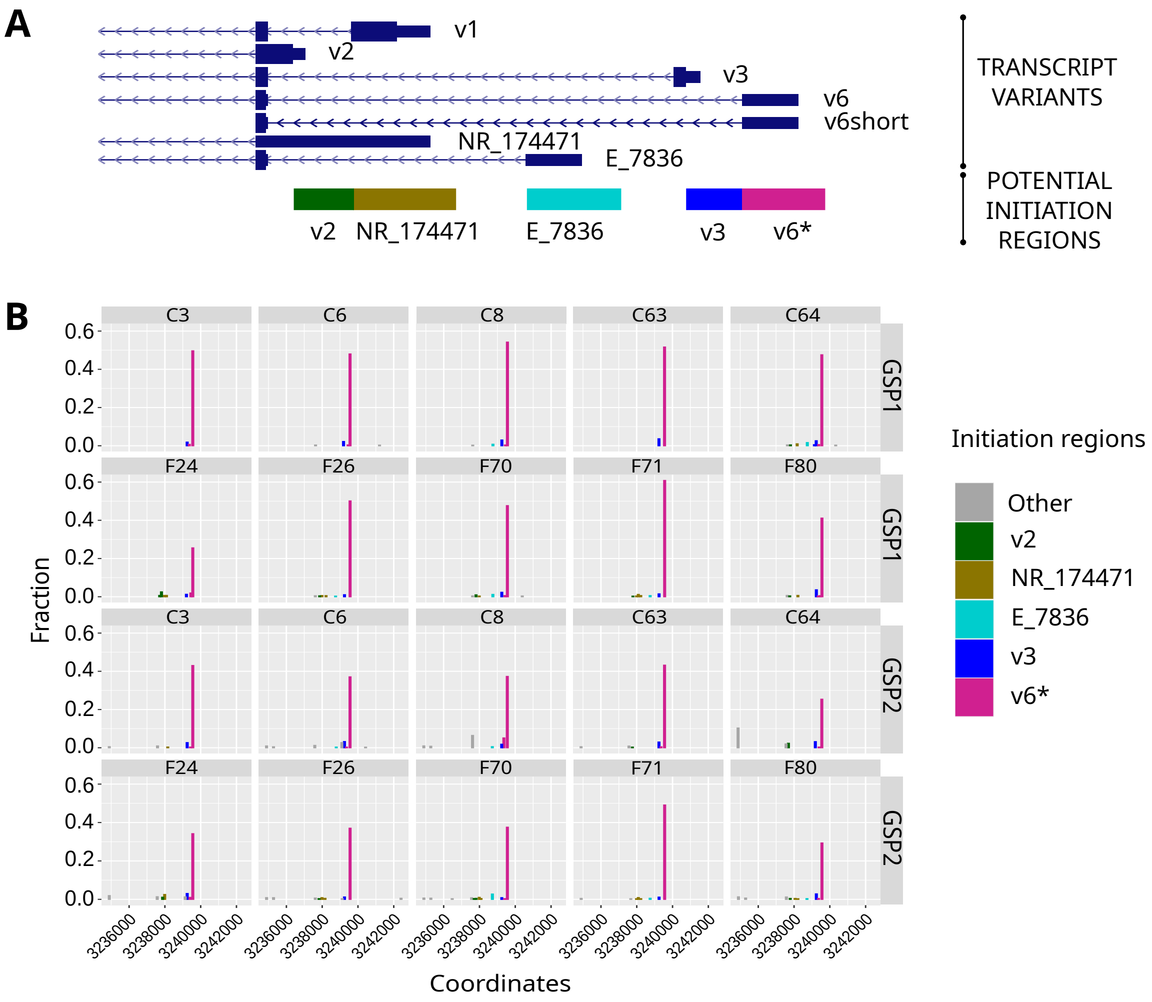

Supplementary Figure S7. Distribution of peaks identified by pracma among potential initiator regions. A. Location of regions in which v2, NR_174471, E_7836, v3 and v6 transcript formation can be initiated. v6* means that the initiation region also corresponds to v6short and GSP1 can detect this transcript. B. Distribution of peaks identified by pracma among these regions.

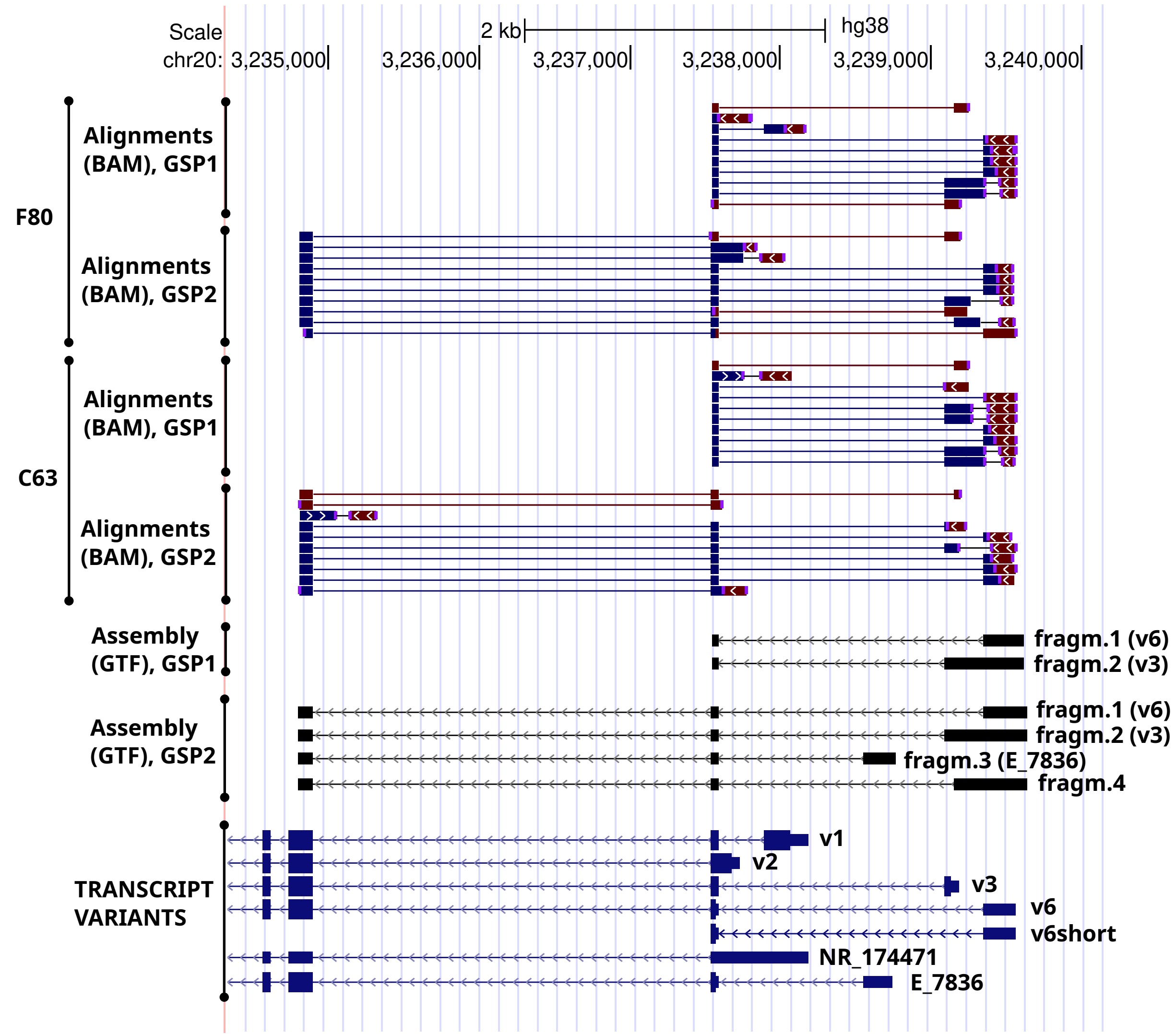

Supplementary Figure S8. *De novo* assembled 5'-ends of transcripts and random alignments from two samples. The first four tracks each show ten random alignments from the files that were used for the assembly of the transcript fragments via StringTie. F80 and C63 are FECD and control samples, respectively, that were randomly chosen.

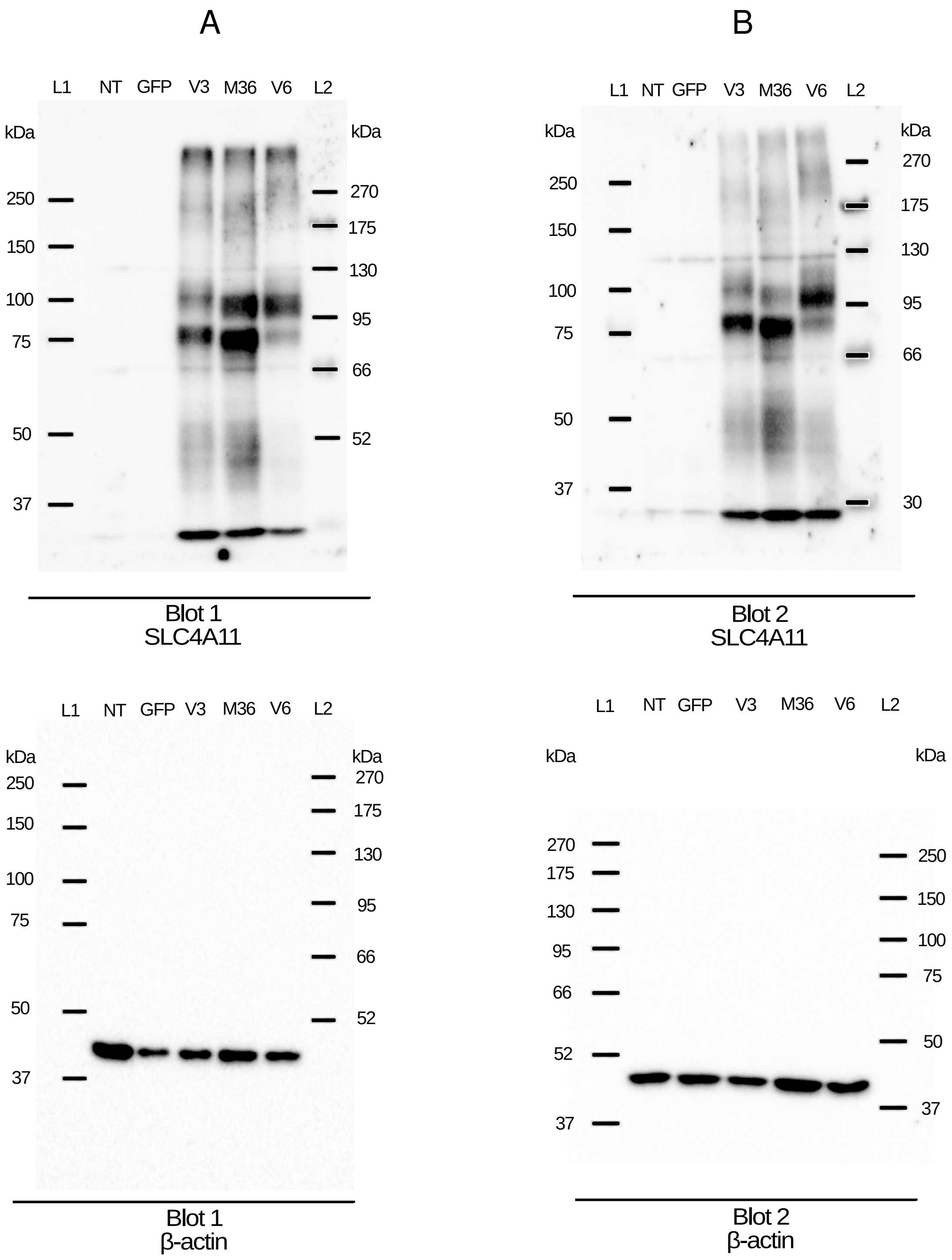

Supplementary Figure S9. Western blot analysis of cell lysates from HEK293 cells transfected with plasmids pV3, pM36, and pV6 (complete membranes). Top panels show immunostaining with anti-SLC4A11 antibodies; bottom panels with anti-β-actin antibodies. L1, L2 – two different protein ladders (see Materials and Methods); NT – no transfection; GFP – transfection with the same plasmid vector as pV3, pM36, and pV6 containing the copGFP gene; v3, M36, v6 – transfection with plasmids pV3, pM36, and pV6, respectively. Panels A and B represent results from two independent transfections.
